# Application of Glycerol for Induced Powdery Mildew Resistance in *Triticum aestivum* L.

**DOI:** 10.3389/fphys.2016.00413

**Published:** 2016-09-21

**Authors:** Yinghui Li, Na Song, Chuanzhi Zhao, Feng Li, Miaomiao Geng, Yuhui Wang, Wanhui Liu, Chaojie Xie, Qixin Sun

**Affiliations:** Key Laboratory of Crop Heterosis and Utilization (MOE) and State Key Laboratory for Agrobiotechnology, Beijing Key Laboratory of Crop Genetic Improvement, China Agricultural UniversityBeijing, China

**Keywords:** *Triticum aestivum* L., glycerol application, induced disease resistance, powdery mildew, G3P, oleic acid, *TaSSI2*, *TaGLI1*

## Abstract

Previous work has demonstrated that glycerol-3-phosphate (G3P) and oleic acid (18:1) are two important signal molecules associated with plant resistance to fungi. In this article, we provide evidence that a 3% glycerol spray application 1–2 days before powdery mildew infection and subsequent applications once every 4 days was sufficient to stimulate the plant defense responses without causing any significant damage to wheat leaves. We found that G3P and oleic acid levels were markedly induced by powdery mildew infection. In addition, *TaGLI1* (encoding a glycerol kinase) and *TaSSI2* (encoding a stearoylacyl carrier protein fatty acid desaturase), two genes associated with the glycerol and fatty acid (FA) pathways, respectively, were induced by powdery mildew infection, and their promoter regions contain some fungal response elements. Moreover, exogenous application of glycerol increased the G3P level and decreased the level of oleic acid (18:1). Glycerol application induced the expression of *pathogenesis-related* (*PR*) genes (*TaPR-1, TaPR-2, TaPR-3, TaPR-4*, and *TaPR-5*), induced the generation of reactive oxygen species (ROS) before powdery mildew infection, and induced salicylic acid (SA) accumulation in wheat leaves. Further, we sprayed glycerol in a wheat field and found that it significantly (*p* < 0.05) reduced the severity of powdery mildew disease and lessened disease-associated kernel weight loss, all without causing any noticeable degradation in wheat seed quality.

## Introduction

Bread wheat (*Triticum aestivum* L.) is one of the most important food crops worldwide. The sustainable production of this crop directly secures national food supplies and affects the economies of many countries. Wheat powdery mildew, caused by the obligate biotrophic ascomycete fungus *Blumeria graminis* f. sp. *tritici* (*Bgt*), infects the aerial parts of wheat and is a devastating disease that affects wheat-growing areas worldwide, with reported yield losses ranging from 14 to more than 30% (Griffey et al., 1993). Genetic resistance and foliar fungicides are available for the control of powdery mildew. However, many resistance genes have lost their effectiveness as new races of the pathogen have evolved. Moreover, fungicide use is not always economically feasible. In this context, the study of the mechanisms of disease resistance and the identification of new ways to improve plant resistance are some of the most important research and development efforts currently underway to increase wheat yields. In response to pathogen attacks, plants have evolved diverse signaling and physiological response pathways. Plant systemic acquired resistance (SAR) is a critically important broad-spectrum immunity response against pathogens. Several compounds that contribute to SAR have been identified, including the nitric oxide (Durner and Klessig, 1999), reactive oxygen species (Durrant and Dong, 2004), the phytohormone salicylic acid (SA) and its methylated derivative MeSA (Park et al., 2007), jasmonic acid (JA; Truman et al., 2007), the dicarboxylic acid azelaic acid (AzA; Jung et al., 2009), auxin (Truman et al., 2010), G3P (Yu et al., 2013; Wang et al., 2014), and some free radicals (Wang et al., 2014), among others.

Glycerol-3-phosphate (G3P), an obligatory precursor in plant glycerolipid biosynthesis, is now recognized as a mobile signal that can induce SAR (Chanda et al., 2011; Yang et al., 2013). Plant G3P is derived from the glycerol kinase (GK)-mediated phosphorylation of glycerol or the NAD-dependent G3P dehydrogenase (G3Pdh)-mediated reduction of dihydroxyacetone phosphate (DHAP) (Figure S1; adapted from Kachroo and Kachroo, 2009). G3P derived from both G3Pdh and GK are known to be important for SAR. Arabidopsis mutations in the genes encoding enzymes related to G3P-synthesis such as *gly1* (a G3Pdh) and *gli1* (a glycerol kinase) reduce the G3P levels and enhance susceptibility to *C. higginsianum* (Chanda et al., 2008; Venugopal et al., 2009). Conversely, Arabidopsis plants overexpressing *GLY1* can increase G3P levels and enhance resistance against *C. higginsianum* (Chanda et al., 2008). Arabidopsis plants overexpressing *GLI1/NHO1* also exhibit increased G3P levels and have enhanced resistance against non-host bacterial (Kang et al., 2003). Moreover, it is known that exogenous glycerol can be absorbed by plant cells, and this can be further phosphorylated to G3P by glycerol kinase (GK) in the cytosol, thereby increasing endogenous G3P levels and enhancing pathogen resistance in Arabidopsis and *Theobroma cacao* (Chanda et al., 2011; Zhang et al., 2015). In wheat, the expression of *TaGLY1* and *TaGLI1* is significantly induced by infection with *Puccinia striiformis* f. sp. *tritici* (*Pst*); G3P levels are also markedly increased in wheat leaves infected with *Pst* race CYR23 (Yang et al., 2013). G3P takes part in NO/ROS → AzA → G3P-induced SAR signaling, which functions in parallel with SA-derived signaling (Yu et al., 2013; Wang et al., 2014).

Several studies have shown that fatty acids (FAs) and their derivatives are associated with a variety of plant responses to both biotic and abiotic stress. Low levels of oleic acid (18:1) results in the alteration of salicylic acid (SA)- and jasmonic acid (JA)-mediated defense responses (Kachroo and Kachroo, 2009; Xia et al., 2009; Savchenko et al., 2010). Low levels of oleic acid can raise NO levels and trigger the induction of the expression of NO responsive nuclear disease-resistance genes (Mandal et al., 2012). Further, free fatty acids (FFAs) can be released by the secreted fungal effector lipase protein (FGL1) during wheat head infection by *Fusarium graminearum*, and the released unsaturated FFAs (e.g., oleic (18:1), linoleic (18:2), and α-linolenic (18:3) acids) can inhibit innate immunity-related callose formation (Blümke et al., 2014). It has been suggested that a high level of unsaturated FAs may be relatively more beneficial to fungi in the infection process while depressed levels of oleic acid are beneficial to plant defense response efforts (Kachroo et al., 2004; Song et al., 2013). Various genetic and molecular techniques can be used to reduce oleic acid levels and thereby increase plant defense responses to pathogens. A mutation in the *SSI2* gene in Arabidopsis that renders the delta-9-stearoyl-acyl carrier protein desaturase (SACPD) unable to convert stearic acid (18:0)-ACP to oleic acid (18:1)-ACP decreases the levels of oleic acid; the *ssi2* mutation exhibits spontaneous lesion formation, severely retards growth, elevates SA and JA levels, induces the expression of *PR* genes, and improves plant resistance to powdery mildew (Kachroo and Kachroo, 2009; Savchenko et al., 2010; Song et al., 2013). Exogenous application of glycerol can increase G3P levels in plants, resulting in a reduction in 18:1 pools via the acylation of G3P with oleic acid, causing *ssi2*-like phenotypes in wild-type plants, resulting in the induction of *PR* gene expression that then confers resistance to fungi in Arabidopsis, rice, soybean, and *Theobroma cacao* (Kachroo et al., 2001, 2004, 2008; Jiang et al., 2009; Zhang et al., 2015).

Many SAR compounds are known to induce plant disease defense programs, such as. SA, Pip, (Me) JA, AzA, ß-aminobutyric acid, and benzo (1,2,3) thiadiazole-7-carbothioic acid S-methyl ester (BTH) (Reimer-Michalski and Conrath, 2016). Among them, BTH is most prominent example; it was introduced commercially to the agrochemical market in 1996 as a so-called “plant activator” with the trade names Bion®, Actigard®, or Boost® (Ruess et al., 1996). Treatment of wheat plants with 30 g of BTH per hectare in repeated field trials led to ~35% reduction in disease symptoms caused by leaf rust fungus compared with the untreated control plants, and led to an increase in yield of ~18% relative to untreated control plants and compared with a 17% yield increase in plants treated with a combination of Tilt (125 g per hectare) and fenpropidin (500 g per hectare) (Görlach et al., 1996). It is reported that disease control of BTH application is not so impressive in the wheat field (Stadnik and Buchenauer, 1999). Acibenzolar-S-methyl (ASM) is shown to control tomato bacterial spot when applied in the field (Obradovic et al., 2004). ASM is also determined to enhance resistance to bacterial wilt pathogen and result in 33 and 13% yield increases in tomato cultivars BHN 466 and Neptune, respectively (Pradhanang et al., 2005). ASM can reduce infection of barley by the leaf scald pathogen *Rhynchosporium secalis* by 45% (Paterson et al., 2008). It is also known that some alterations in primary metabolism can cause plants to induce tissue-specific immunity responses to pathogens. For instance, application of sugar to plants has been shown to activate the expression of antimicrobial pathogenesis-related genes (Herbers et al., 1996). Glycerol is a non-toxic and environmentally-friendly sugar alcohol (Yang et al., 2012). The potential for using glycerol to improve plant disease resistance responses clearly merits further study. To date, there have been no reports of glycerol-induced pathogen resistance in wheat. In this study, we found that the exogenous application of glycerol induced pathogen resistance responses in wheat. We measured the G3P and oleic acid (18:1) levels in wheat leaves during infection with powdery mildew, and observed alterations in the expression of genes associated with the glycerol and FA pathways. We also evaluated the expression of genes in some basal signaling resistance pathways in wheat leaves following glycerol application. Finally, we sprayed glycerol in a wheat field and found that glycerol has some potential for use as an environmentally safe foliar spray to protect wheat against powdery mildew.

## Materials and methods

### Plant and fungal growth conditions and pathogen inoculations

Seed stocks for the susceptible wheat lines Xuezao, Chinese spring, Jimai5265, Liaochun10, and durum wheat Mo75 (susceptible to wheat powdery mildew), Jing411 (partially-resistant to wheat powdery mildew), and the four resistant wheat lines 6Y8, 6Y9, 6Y16, and 6Y19 (carrying resistant gene *Pm21*) are maintained in our laboratory. Powdery mildew isolate E09 was provided by Prof. Xiayu Duan of the Institute of Plant Protection of the Chinese Academy of Agricultural Sciences in Beijing. Wheat was cultivated in a growth chamber at a relative humidity of 75% and 26/20°C day/night temperature regime with a 14/10 h (light/dark) photoperiod and a light intensity of 3000 lx. Powdery mildew fungus isolate E09 was maintained on the susceptible genotype wheat line Xuezao by weekly transfer of conidia to new plants. Seedlings of wheat lines were artificially inoculated by dusting sufficient amount of *Bgt* E09 conidia from sporulating seedlings of “Xuezao” at the stages when the first leaf was fully expanded, it was difficult to calculate and control the concentration of spores, so the sufficient amount of spores were needed to make sure wheat leaves was infected with enough powdery mildew in their two surfaces; mock inoculation plants were not inoculated by any conidia. The germination/penetration rates of conidiophores (number of germinated spores or penetration spores relative to the total number of spores in 5 cm long leaf segments) were calculated and presented as the means of three independent replicated experiments. In each independent experiment, 15–20 leaf segments were observed at 12, 24, 48, and 96 h post-powdery mildew infection (hpi).

### Glycerol treatments and field trials

**Figure 1:**
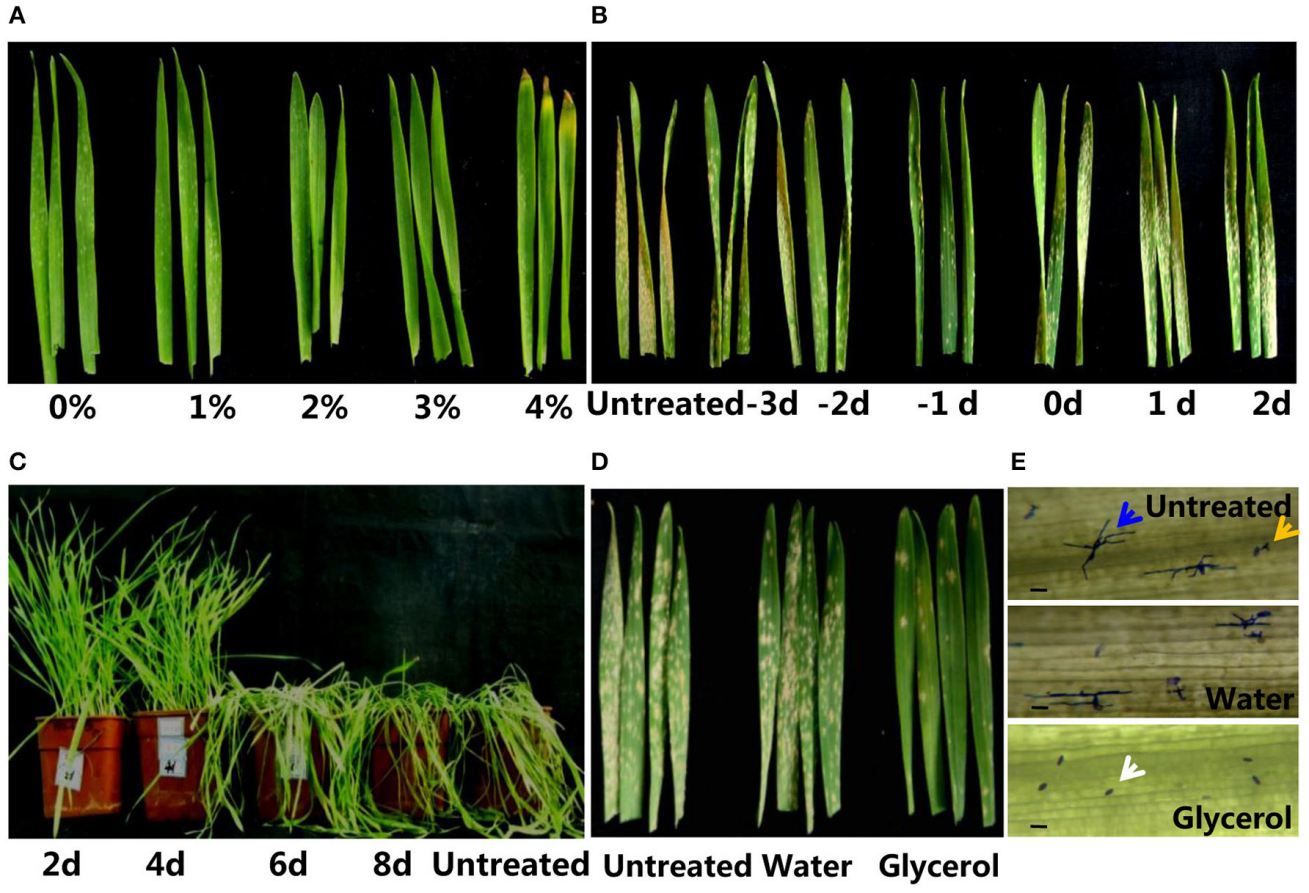
**Application of glycerol enhanced powdery mildew resistance in wheat. (A)** Morphology of Xuezao leaves at 1 week post-glycerol application (0, 1, 2, 3, and 4% glycerol solutions). **(B)** Resistance phenotypes of Xuezao leaves with 3% glycerol treatment applied at a series of timings prior to and after powdery mildew inoculations. (−3−2) Glycerol treatments at −3, −2, −1, 0, 1, 2 days before (−) and after powdery mildew inoculation. The resistance phenotypes were observed at 2 weeks post-powdery mildew infection. **(C)** Resistance phenotypes of plants with differing frequencies glycerol treatment at 3 weeks post-powdery mildew infection. (2–8 days) The plants treated with glycerol once every 2, 4, 6, or 8 days. **(D)** Images of untreated, water-treated, and glycerol-treated Xuezao leaves post-powdery mildew-infection with growth of 2 weeks under optimized conditions. **(E)** Morphology of conidiophores in water-treated and glycerol-treated Xuezao leaves at 48 h post-powdery mildew-infection (hpi). Penetration spores that produced secondary hyphae are indicated by blue arrows; germinated spores are indicated by yellow arrows; the white arrows indicate spores that did not germinate. Bars = 100 μm. (Untreated) Xuezao leaves without water or glycerol treatments; (Water) Xuezao leaves with water treatments; (Glycerol) Xuezao leaves with glycerol treatments.

Glycerol treatments were performed by spraying 1, 2, 3, and 4% glycerol solutions prepared in sterile water containing 0.02% Silwett L-77 (Fisher Scientific, Cat. NC0138454, this treatment was employed for the experiment depicted in Figure 1A). The 3% glycerol treatment was subsequently selected as the optimized treatment condition and this used as the concentration in most of the glycerol spraying experiments. Water containing 0.02% Silwett L-77 was used as the control treatment. At the seedling stage (when the first leaf was fully expanded), both the water and the glycerol solutions were sprayed (“pretreatment of plants”) onto wheat leaves until liquid was dripping from the leaves; the untreated plants were without water or glycerol pretreatments. One day after the pretreatment, wheat plants were inoculated with a copious amount of spores of a locally-prevalent *Bgt* isolate E09 or spores of leaf rust isolate PHT, and these plants were spayed one time each 3 days from the time of the first glycerol or water treatment (this treatment was employed for the experiments depicted in Figure 1D, Figures S3C, S4, and the resistance phenotypes of plants were observed at 2 weeks post-powdery mildew infection; this period included a total of four glycerol applications). For the adult stage spraying treatments, the 3% glycerol treatment, water treatment or untreated group (without water or glycerol treatments) were implemented in two field plot experiments. In the artificial inoculation field experiments, where powdery mildew and leaf rust were inoculated repeatedly, the Xuezao seedlings with sporulating powdery mildew (prepared in greenhouse) were evenly transplanted in the spreader rows of Xuezao throughout the field as the overwinter wheat seedlings had recovered the spring growth (15 Apr 2015; 13 Apr 2016, Beijing, China). The transplanted inoculum plants were used as the source to provided spores to infect plants adjacent to these “spreader” rows, thereby relaying the inoculation process, leading ultimately to an outbreak of powdery mildew throughout the field. The development of powdery mildew on wheat plants occurred from the bottom up to the flag leaves and spikes. The leaf rust disease inoculation process comprised the injection by syringe of a suspension of rust spores (with 0.05% Tween 20) into the plants of spreader rows. The outbreak of leaf rust generally occurred later than the powdery mildew outbreaks. The naturally occurring infection field trials were conducted 30–50 m away from the artificial inoculation field trials (so these shared the same environmental conditions). The infection of in the naturally occurring infection field trials resulted from the spread of spores from the artificial inoculation field. The outbreak of disease in the naturally occurring infection field occurred later than, and was not as serious as, the outbreak in the artificial inoculation fields. The water or the 3% glycerol solution were sprayed before the outbreak of powdery mildew disease (when the infection was on the lower part of plant and had not developed up to flag leaves), which occurred around the booting and flowering stages (10 May 2015; 8 May 2016) in the artificial inoculation field, and these plants were spayed one time each 3 days from the time of the first glycerol or water treatments; this period included a total of four glycerol or water applications. In the natural infection field, the glycerol applications were performed later (15 May 2015; 13 May 2016) than in the artificial inoculation field, and this period included a total of two glycerol or water applications. The effect of the glycerol treatments was observed 5 days after the final application (the treatments were employed for the experiments depicted in **Figures 5D**, **6**, **7**, Figures S3D, S6–S10). The wheat in the field plot was planted via mechanized seeding; we sampled from a field with the same plant density, using a randomized block design, with three independent replications. Each field plot area was about 0.4 m^2^ (1 m^*^0.4 m, containing about 400 ears of wheat).

### Cloning of genes, promoter sequence analysis, and prediction of cis-regulatory elements

The large sequence fragments containing the *TaSSI2* (IWGSC_ chr2AL_ab_k71_contigs_longerthan_200_6380845, 10387 bp), *TaGLI1* (IWGSC_chr2DL_ab_k71_contigs_longerthan_200_9840377, 6269 bp) and *TaGLY1* (IWGSC_chr4DL_V3_ab_k71_contigs_longerthan_200_14463426, 20840 bp) were obtained from IWGSC (http://www.wheatgenome.org), subjected to BLAST analysis using the query sequences AK332689.1, KC244204.1, and KC527592.1, which were predicted to be the as homologs of, respectively, *AtSSI2* (At2g43710), *AtGLI1* (At1g80460), and *AtGLY1* (At2g40690). The *TaGLI1* genes were cloned based on the large sequence fragments with a homologous cloning strategy. The primers were designed using DNAMAN software. Putative stress and hormone responsive cis-regulatory elements were predicted and analyzed using Softberry (http://linux1.softberry.com/berry.phtml) and SOGO (https://sogo.dna.affrc.go.jp). The expression patterns of target genes were searched from the Plant Expression Database (http://www.plexdb.org/index.php).

### RNA extraction and RT-PCR

The Xuezao leaves were treated with water or glycerol at the seeding stage when the first leaf was fully expanded. One day after the pretreatment, wheat plants were inoculated with *Bgt* isolate E09, and the leaves tissues from both the water-treatment and glycerol-treatment were collected at 0, 12, 24, and 48 hpi (this period included a total of one glycerol application), and frozen in liquid nitrogen. Total RNA was extracted using an RNA pure Plant Kit (TIANGEN). cDNA was synthesized from total RNA using a PrimeScript™ RT reagent Kit and oligo (dT) primers (Takara). The gene-specific primers of the target genes and *Actin* gene were designed using DNAMAN software; the primer sequences are listed in Table S1. All PCR amplifications were performed in a volume of 20 μl containing 10 μl FastStart Universal SYBR Green Master (Roche). PCR amplification was conducted with an Applied Biosystems 7500 Real-Time PCR System (ABI, USA). PCR conditions consisted of an initial step at 95°C for 3 min followed by 40 cycles of 95°C for 15 s, 59°C for 15 s, and 72°C for 30 s. The expression levels of target genes were calculated using the 2^−ΔΔCt^ method, differences between the Ct values of a given target gene and *Actin* were calculated as ΔCt = Ct_targetgene_− Ct_Actin_, and the relative expression levels of target genes were determined as 2^−ΔΔCt^. All reactions were performed in triplicate, and each experiment included three independent biological repetitions.

### Measurement of endogenous G3P, fatty acid, and free SA content

The Xuezao leaves were treated with water or glycerol at the seeding stage. One day after the pretreatment, the inoculation plants were inoculated with *Bgt* isolate E09; the mock-inoculation plants were without powdery mildew infection. The leaves tissues from both the water-treatment and glycerol-treatment were collected at 24 hpi, the mock-inoculation leaves tissues were also collected at the same time for the measurement of endogenous G3P, fatty acid, and free SA content.

To determine G3P levels, 3g of wheat leaf tissue was frozen in liquid nitrogen and ground to a fine powder. About 30 mg of ground tissue was weighed and extracted with 5 mL of 80% (v/v) ethanol and centrifuged at 5520 × g for 5 min at 4°C. The supernatants were collected, and 10 μL aliquots were analyzed by LC-MS (Thermo Scientific). The method and instrumentation were based on an ion-pairing reversed-phase negative ion Orbitrap method; An Agilent column was used (Poroshell 120 EC-C18 3.0^*^75 mm 2.7-Micron). The chromatography was performed with a WatersAcquity UPLC I-Class pump (Waters) with a flow rate of 0.2 mL/min. The spray voltage for the ion source used was 3.0 kv. The integrated areas of the reconstructed ion chromatograms for the analyte ion (based on authentic reference standard data) were used to measure the amount of G3P in the extracts (Zhang et al., 2015).

An HP6890 gas chromatogram (GC) (Agilent Technologies, USA) was used for the fatty acid analysis. Tissues were dried in an oven at 45°C for 60 h and then ground into a powder. 200 mg of powder for each sample was placed in a screw capped glass vial and 3 ml of methyl alcohol: acetyl chloride (5:1) was added, followed by 3 ml of hexanes containing an internal reference compound (methyl non-adecanoate, 1 mg/ml, Sigma). After vortexing for 1 min, the samples were heated for 2 h in a water bath at 80°C. After the contents were cooled to room temperature, 4 ml of 7% K_2_CO_3_ was added to neutralize the extracted fatty acids, and the upper phase was transferred to clean sample vials. Samples at 250°C were automatically injected (1 μl) and separated in the GC system equipped with a HP-INNOWAX polyethylene glycol capillary column (30 m × 320 μm × 0.5 μm, Agilent Technologies). The GC was operated at constant flow pressure of 140.9 kPa with an initial oven temperature of 220°C. The initial step of the method was isothermal for 13 min, followed by a temperature ramp of 20°C/min to 240°C and then isothermal for 5 min. The FID temperature was 250°C, and the split ratio of nitrogen was 20:1. Fatty acids were identified by comparison of their retention times with the retention time of the internal reference compound. All data were analyzed with ChemStation software (Agilent Technologies) and were normalized to sample weight and the internal reference (Yang et al., 2010). SA was extracted and measured from leaf tissue (~0.3 g of fresh weight), as described previously (Segarra et al., 2006).

### Coomassie blue, DAB staining

The *Bgt*-infected leaves were depigmented in 75% ethanol solution for 24 h at 30°C, then moved into staining solution [a 1:1 (v:v) mixture of coomassie blue R250 (0.6% (w/v)] in methanol, and 0.5% trichloroacetic acid in water) for 5 min and cleared immediately in sterile water. DAB staining solution (0.1 g DAB, 100 ml distilled water, KOH adjust pH = 5.8) was used to stain infected leaves for 8 h at 28°C. 100% ethanol was used to depigment infected leaves for 24 h. Each experiment included three independent biological repetitions, and 15–20 leaves were observed in each independent biological experiment.

### SDS sedimentation volume and protein content determination

SDS sedimentation volume measurements were performed with a rapid analytical method to assess gluten strength (Axford et al., 1979). Whole-meal flour equivalent to 6 g (at 15% moisture content) from each sample was suspended in 50 ml of distilled water in a 100-ml cylinder before the addition of 50 ml of SDS reagent (20 g SDS+20 ml diluted lactic acid in a liter of distilled water) and further suspension. Sediment volumes were recorded after 20 min of settling. Grain protein content (GPC) was measured by near-infrared reflectance spectroscopy (NIRS) on a Perten DA-7200 instrument (Perten Instruments) and expressed on a 14% moisture basis.

### Statistical analysis

Analysis of variance (ANOVA) was performed to evaluate the statistical significance of differences between each treatment using SPSS. Fisher's LSD multiple range tests were used for multiple comparison tests. *P* < 0.05 was considered significant.

## Results

### Glycerol application enhanced powdery mildew resistance in wheat

To examine whether glycerol could induce resistance responses in wheat and to determine the optimal concentrations and timing of glycerol application, we sprayed a series of concentrations of glycerol on the susceptible wheat line Xuezao and examined plant resistance to powdery mildew. The resistance levels increased to differing degrees when sprayed with the 1–4% glycerol solutions; the germination rates of conidiospores decreased significantly as the glycerol concentration increased (Figure S2), though the 4% glycerol solution reesuled in leaf tip necrosis (Figure 1A). To evaluate the optimal duration of glycerol treatment for effective enhancement of disease resistance, 3% glycerol treatments were applied at a series of timings prior to and after powdery mildew inoculations. The result showed that application of glycerol 1–2 days before inoculation of powdery mildew effectively induced disease resistance; when applied on the same day as the inoculation of fungus or thereafter, glycerol did not cause effective resistance in plants (Figure 1B). With continuous powdery mildew inoculation in the greenhouse, durable resistance required spraying plants with glycerol at least once every 4 days (Figure 1C). Under the optimized conditions (3% glycerol treatment applied 1 day prior to powdery mildew infection, and once each 3 days following the first glycerol treatment), glycerol application induced disease resistance at the seedling stage and also increased resistance in adult plants (Figure 1D and Figure S3D). At 2 weeks post-powdery mildew-infection, both untreated and water-treated control plants were highly susceptible, while the glycerol-treated plants were highly resistant to powdery mildew (Figure 1D). Germination of wheat seeds in 2.5–3% glycerol also induced resistance at the seedling stage, but root and shoot growth were obviously inhibited by this glycerol treatment (Figures S3A,B). We also found that glycerol application could enhance powdery mildew resistance in different wheat varieties (Chinese spring, Jimai5265, Liaochun 10, and durum wheat Mo75) (Figure S3C). We carried out coomassie brilliant blue staining to monitor the growth of conidiophores in the diseased leaves that had been treated with glycerol or water. The germination rates and penetration rates of powdery mildew on glycerol-treated leaves were remarkably lower than those of the untreated and the water-treated leaves at 12, 24, 48, and 96 h post-infection (Table S2, the morphology of conidiophores in untreated, water-treated, and glycerol-treated Xuezao plants at 48 hpi is shown in Figure 1E). We also observed that glycerol application slowed the spread of leaf rust infection in the leaves of wheat seedlings (Figure S4).

### Altered G3P levels were associated with wheat resistance to powdery mildew, and glycerol application increased G3P levels

**Figure 2:**
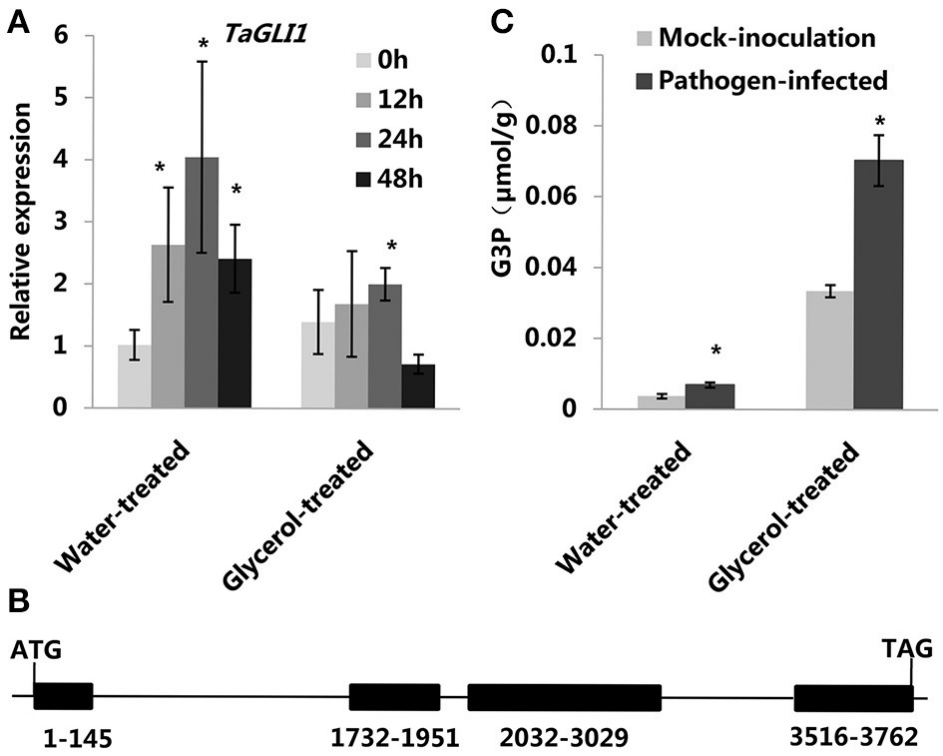
**Altered ***TaGLI1*** expression and G3P levels were associated with wheat resistance to powdery mildew**. **(A)** Relative transcriptional changes of *TaGLI1* in water-treated (control) and glycerol-treated Xuezao leaves at 0, 12, 24, and 48 hpi. Each value is the mean ± SE of three independent biological repetitions. Asterisks indicate significant differences from water-treated (0 hpi) samples at *P* < 0.05 by Student's *t*-test. **(B)** The structure of the *TaGLI1* gene. **(C)** Changes of G3P levels in response to powdery mildew infection in both the water-treated and the glycerol-treated Xuezao leaves. (Pathogen-infected) Leaf tissues were sampled at 24 h post-powdery mildew-infection; (Mock-inoculation) Leaf tissues were sampled without powdery mildew-infection. Each value is the mean ± SE of three independent biological repetitions. Asterisks indicate significant differences from mock-inoculation samples at *P* < 0.05 by Student's *t*-test.

Plant G3P homeostasis is known to be regulated in part by two genes: *GLI1* (encoding a glycerol kinase) and *GLY1* (encoding a G3P dehydrogenase). We therefore examined the expression patterns of *TaGLI1* (GenBank: KC244204.1) and *TaGLY1* (GenBank: KC527592.1) in the data from a previous microarray experiment (Figures S5B,C; Xin et al., 2011). We found that only *TaGLI1* had up-regulated expression following powdery mildew infection; *TaGLI1* expressoin increased in both the susceptible variety Jingdong 8 and in itsresistant near-isogenic line (NIL) Pm30 (carrying the resistance gene *Pm30*). To verify the microarray results, we used RT-QPCR analysis to measure the expression of *TaGLI1* and *TaGLY1* both in the water-treated (control) and glycerol-treated leaves at 0, 12, 24, and 48 hpi. The expression of *TaGLI1* increased after infection with powdery mildew (Figure 2A); it increased by ~4-fold at 24 hpi in the water-treated leaves, but was only increased by about 2-fold in the glycerol-treated leaves (Figure 2A). In contrast, the expression of *TaGLY1* showed little change in response to powdery mildew infection (Figure S5A). We cloned the *TaGLI1* gene of the susceptible line Xuezao with a homologous cloning strategy. A sequence comparison between the PCR amplification products from experiments using cDNA (1605 bp) or genomic DNA (3762 bp) as the template revealed that *TaGLI1* has 4 exons and 3 introns (Figure 2B). We used Softberry and the SOGO database to analyze the sequence and identified putative stress and hormone responsive cis-regulatory elements 1.5 kb upstream of the transcription start site (TSS) of *TaGLI1*. WBOXNTERF3, GT1GMSCAM4, MYCATRD22, GT1CONSENSUS, MYCCONSENSUSAT, WRKY71OS, and WBOXATNPR1 elements were found in *TaGLI1* promoter region. These cis-regulatory elements are known to mediate stress responses through the gibberellic acid, ABA, and salicylic acid pathways (Table 1). However, we found only two predicted fungal response elements (2 instances of the GT1CONSENSUS) in the *TaGLY1* promoter region. Given that the *TaGLI1* gene had markedly increased expression within 24 h of powdery mildew inoculation, we monitored the G3P levels at 24 hpi. Compared to the mock-inoculation plants, the pathogen-infected plants had ~2-fold higher levels of G3P both in the water-treated (control) and the glycerol-treated leaves. Glycerol treatment resulted in ~10-fold higher levels of G3P in the glycerol-treated leaves as compared to water-treated leaves (Figure 2C). The high level of G3P increased by glycerol application might contribute to wheat powdery mildew resistance.

**Table 1:** **The predicted stress-responsive ***cis-elements*** present in the ***TaGLI1*** and ***TaSSI2*** promoter regions**.

***Cis-elements***	**Core sequence**	***TaGLI1* promoter**	***TaSSI2* promoter**	**Role**
WRKY71OS	TGAC	9	3	A transcriptional repressor of the gibberellin signaling pathway (Zhang et al., 2004), Parsley WRKY proteins bind specifically to TGAC-containing W box elements within the *Pathogenesis-Related Class10* (*PR-10*) genes (Eulgem et al., 1999).
WBOXATNPR1	TTAGC	1	2	“W-box” found in the promoter of the *AtNPR1* gene recognized specifically by salicylic acid (SA)-induced WRKY DNA binding proteins (Yu et al., 2001).
MYCCONSENSUSAT	CANNTG	4	4	MYC recognition site found in the promoters of the dehydration-responsive *rd22* gene and many other genes in Arabidopsis (Abe et al., 2003).
MYCATRD22	CACATG	1	0	MYC binding site in the *rd22* gene of *Arabidopsis thaliana*; ABA-induction (Abe et al., 1997).
GT1GMSCAM4	GAAAAA	1	0	Plays a role in pathogen- and salt-induced *SCaM-4* gene expression (Park et al., 2004).
GT1CONSENSUS	GRWAAW	4	1	Binding of GT-1-like factors to the *PR-1a* promoter influences the level of SA-inducible gene expression (Buchel et al., 1999).
WBOXNTERF3	TGACY	1	2	May be involved in activation of the *ERF3* gene in response to wounding (Nishiuchi et al., 2004).

### Altered fatty acid levels were associated with wheat resistance to powdery mildew, and glycerol application reduced oleic acid levels

**Figure 3:**
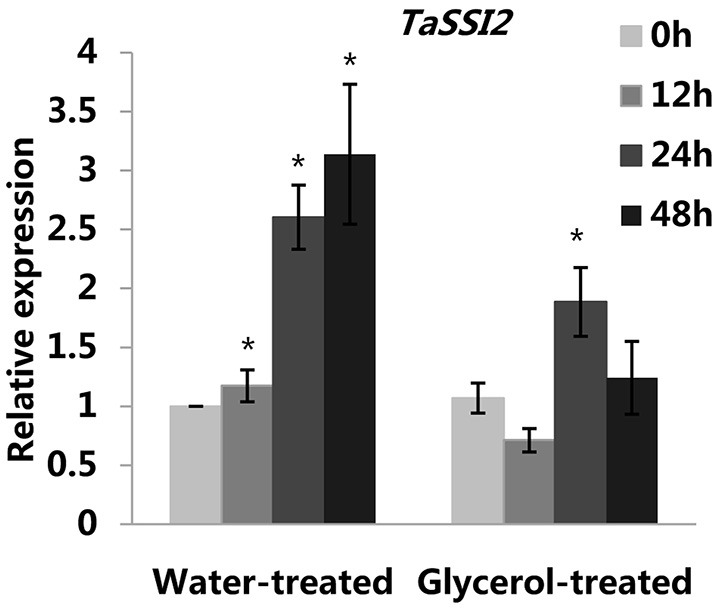
**Relative transcriptional changes of ***TaSSI2*** in the water-treated and glycerol-treated Xuezao leaves at 0, 12, 24, and 48 hpi**. Each value is the mean ± SE of three independent biological repetitions. Asterisks indicate significant differences from water-treated (0 hpi) samples at *P* < 0.05 by Student's *t*-test.

**Figure 4:**
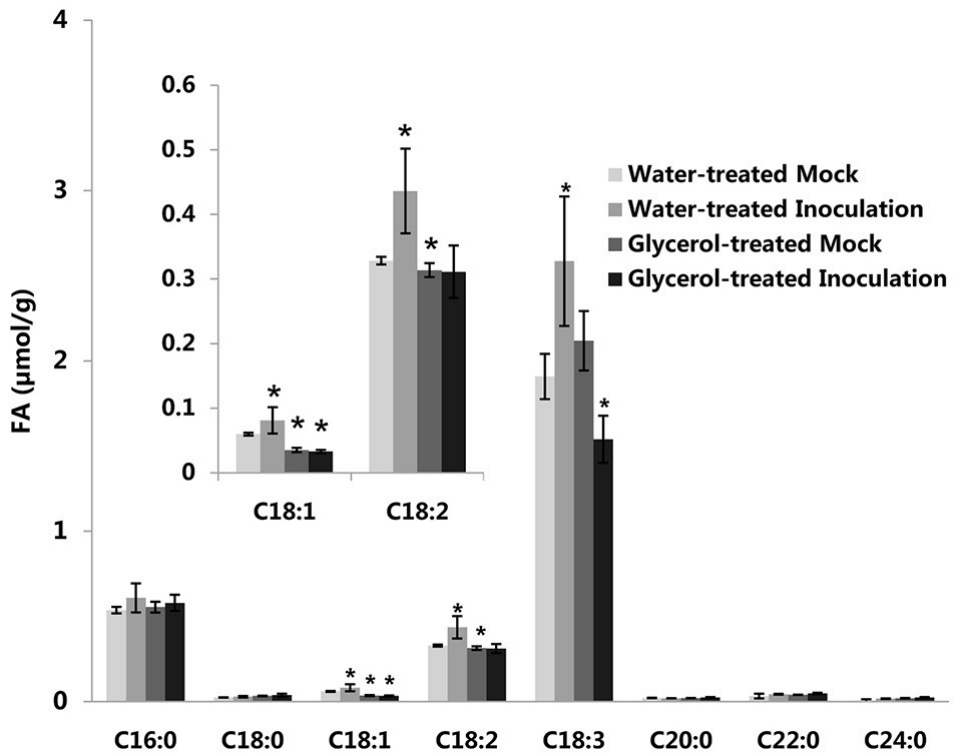
**Changes of FA levels in response to powdery mildew inoculation in both the water-treated and in the glycerol-treated Xuezao leaves**. (Inoculation) Leaf tissues were sampled at 24 hpi; (Mock) Leaf tissues were sampled without powdery mildew-infection. Each value is the mean ± SE of three independent biological repetitions. Asterisks indicate significant differences from water-treated mock samples at *P* < 0.05 by Student's *t*-test.

Previously, we reported that the expression of the *TaSSI2* gene (GenBank: AK332689) was up-regulated in the susceptible variety Jingdong 8 at 12 hpi, while no significant differences were observed in the expression of this gene in the near-isogenic resistant line Pm30 (Song et al., 2013). This result was in accord with our previous microarray analysis (Figure S5D; Xin et al., 2011). It has been shown that the overexpression of the *TaSSI2* gene in the Arabidopsis *ssi2* mutant can compromise its resistance to powdery mildew through increasing oleic acid levels (Song et al., 2013). Therefore, we measured the expressions of *TaSSI2* in the water-treated and glycerol-treated Xuezao leaves at 0, 12, 24, and 48 hpi to examine the role of *TaSSI2* in glycerol-induced powdery mildew resistance. The expression of the *TaSSI2* gene increased in the water-treated leaves 12, 24, and 48 hpi, while the expression of *TaSSI2* showed less change in the glycerol-treated leaves at any of the post-infection time points than in the water-treated leaves (Figure 3). We used Softberry and the SOGO database to analyze the sequence of *TaSSI2* gene, and identified some putative hormone, stress, and fungal responsive cis-regulatory elements 823 bp upstream of the TSS of *TaSSI2* (Table 1). Next, we measured the changes in FA levels in response to powdery mildew infection at 24 hpi. In the water-treated leaves, unsaturated fatty acid levels (18:1, 18:2, 18:3) were increased in pathogen-infected leaves as compared with the mock-inoculation leaves. There were no changes in the levels of saturated fatty acids (16:0, 18:0, 20:0, 22:0, 24:0) (Figure 4). However, in the glycerol-treated leaves, the unsaturated fatty acid levels (18:1, 18:2, 18:3) did not increase in response to powdery mildew infection. Glycerol application lowered the oleic acid level in both the mock-inoculation and the pathogen-infected leaves, as compared with the water-treated leaves (Figure 4). The reduced oleic acid levels in the glycerol-treated plants might be related to wheat powdery mildew resistance.

### Glycerol induced the expression of *PR* genes and increased the accumulation of both reactive oxygen species (ROS) and salicylic acid (SA) in wheat leaves

**Figure 5:**
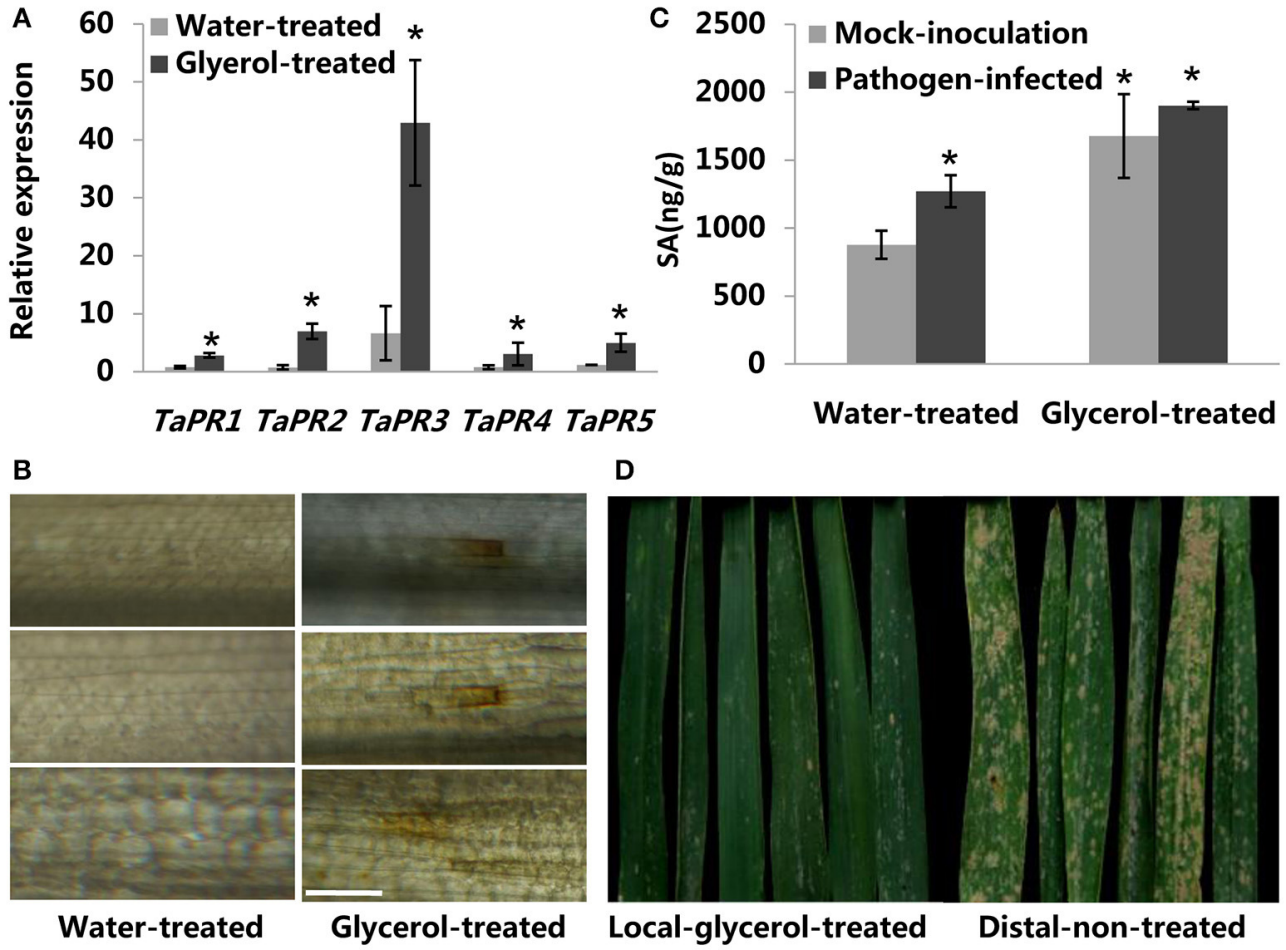
**Glycerol-mediated resistance pathways in wheat. (A)** Relative transcriptional changes of *pathogenesis-related* (*PR*) genes in water-treated and glycerol-treated leaves. Leaf tissues were sampled at 24 h post-glycerol treatments. Each value is the mean ± SE of three independent biological repetitions. Asterisks indicate significant differences from water-treated samples at *P* < 0.05 by Student's *t*-test. **(B)** (ROS) Images of water-treated and glycerol-treated leaves infiltrated with DAB; leaves were observed at 24 h post-glycerol treatment under fluorescence microscopy. Bars = 100 μm. **(C)** Changes of SA levels in response to glycerol application and powdery mildew-infection. (Pathogen-infected) Leaf tissues were sampled at 24 h post-powdery mildew-infection; (Mock-inoculation) Leaf tissues were sampled without powdery mildew-infection. Each value is the mean ± SE of three independent biological repetitions. Asterisks indicate significant differences from mock-inoculation samples at *P* < 0.05 by Student's *t*-test. **(D)** Resistance phenotypes of local-glycerol-treated and distal-non-treated adult-stage Xuezao leaves in the artificial inoculation field. Local-glycerol-treated leaves were treated with glycerol; distal-non-treated leaves had no glycerol treatment.

Previous reports have indicated that multiple *pathogenesis-related* (*PR*) genes (e.g., *PR-1, PR-2, PR-3, PR-4*, and *PR-5*) take part in direct antifungal and/or anti-oomycete activities in various plant species (Yun et al., 1997; Castillo et al., 2005; van Loon et al., 2006). Therefore, we analyzed the expression of several *TaPR* genes in glycerol-treated and water-treated leaves that did not were no infected with powdery mildew. The expression of the following genes was induced in the glycerol-treated leaves: *TaPR-1* (acidicproteins; Lu et al., 2011), *TaPR-2* [(1,3)-β-glucanase; Shetty et al., 2009], *TaPR-3* (chitinase; Shetty et al., 2009), *TaPR4* (wheatwin; Bertini et al., 2006), and *TaPR-5* (thaumatin-like protein; Wang et al., 2010). The expression of these *PR* genes was as least as 3.6-fold greater in the glycerol-treated leaves than in water-treated leaves, and *TaPR-2* expression was almost 10-fold greater in glycerol-treated leaves than in water-treated leaves (Figure 5A). The high expression levels of *TaPR* genes induced by glycerol application before powdery mildew infection might contribute to wheat resistance. Numerous studies have shown that the production and accumulation of ROS and SA in infected plant tissues play important roles in early pathogen recognition and plant innate immunity (Dat et al., 2000; Park et al., 2007; Schwessinger and Ronald, 2012). Therefore, we measured the levels of ROS in water- and glycerol-treated leaves with a DAB staining method (Fryer et al., 2002). Glycerol application induced ROS generation in wheat leaves prior to powdery mildew-infection, while water application did not induce any ROS generation (Figure 5B). Both powdery mildew infection and glycerol application resulted in increased SA levels in wheat leaves (Figure 5C).

### Glycerol application induced local resistance in wheat

In order to test whether glycerol treatment induces whole plant resistance or just induces local resistance. We spayed glycerol on particular (local) leaves and found that such an application did not induced resistance in distal non-treated leaves (Figure 5D). In addition, we applied glycerol on one half of a leaf and monitored the resistance of the whole leaves. Glycerol application to part of a leaf did not confer resistance to the whole leaf; we noticed that spaying glycerol on the top half of leaves did not induce any resistance in the bottom half of leaves but that spaying glycerol on the bottom half leaves seems to have induced some minor little resistance in the top half of leaves (Figure S6). We speculate that glycerol may be absorbed and transported short-distances through water transportation in leaves. We also found that spraying glycerol on wheat ears induced high resistance in these organs, while spraying glycerol only on the leaves did not induce resistance in wheat ears (Figure S10A). We therefore conclude that glycerol application induces localized resistance in wheat.

### The potential of glycerol application as a foliar spray in field conditions

**Figure 6:**
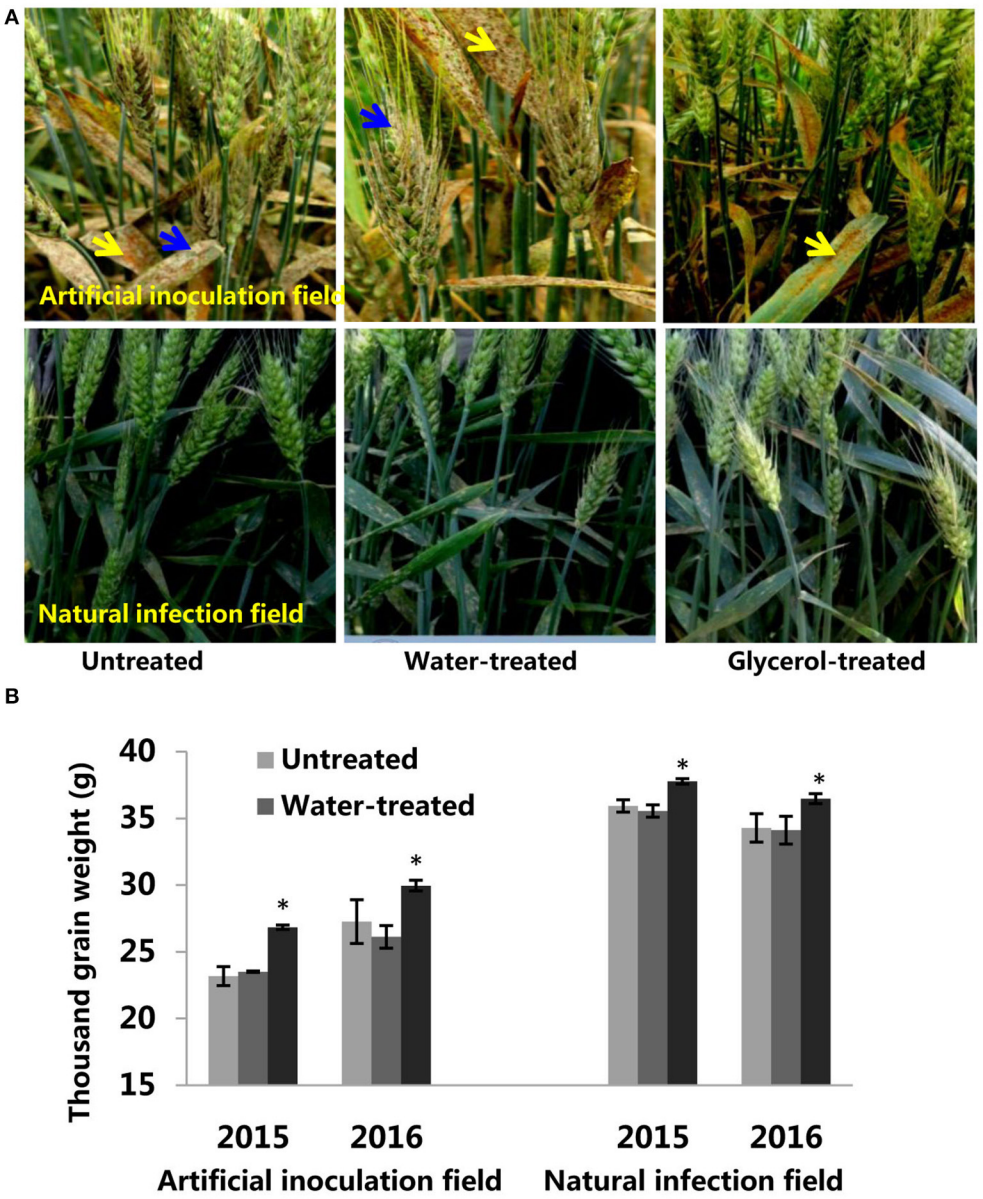
**The potential of glycerol application as a foliar spray in field conditions. (A)** Images of untreated, water-treated, and glycerol-treated Xuezao plants in the artificial inoculation field and natural infection field. Powdery mildew disease symptoms are indicated by blue arrows; Leaf rust disease symptoms were indicated by yellow arrows. **(B)** The thousand kernel weight of untreated, water-treated, and glycerol-treated Xuezao plants in the artificial inoculation field and natural infection field. Each value is the mean ± SE of three independent biological repetitions. Asterisks indicate significant differences from untreated samples at *P* < 0.05 by Student's *t*-test.

**Figure 7:**
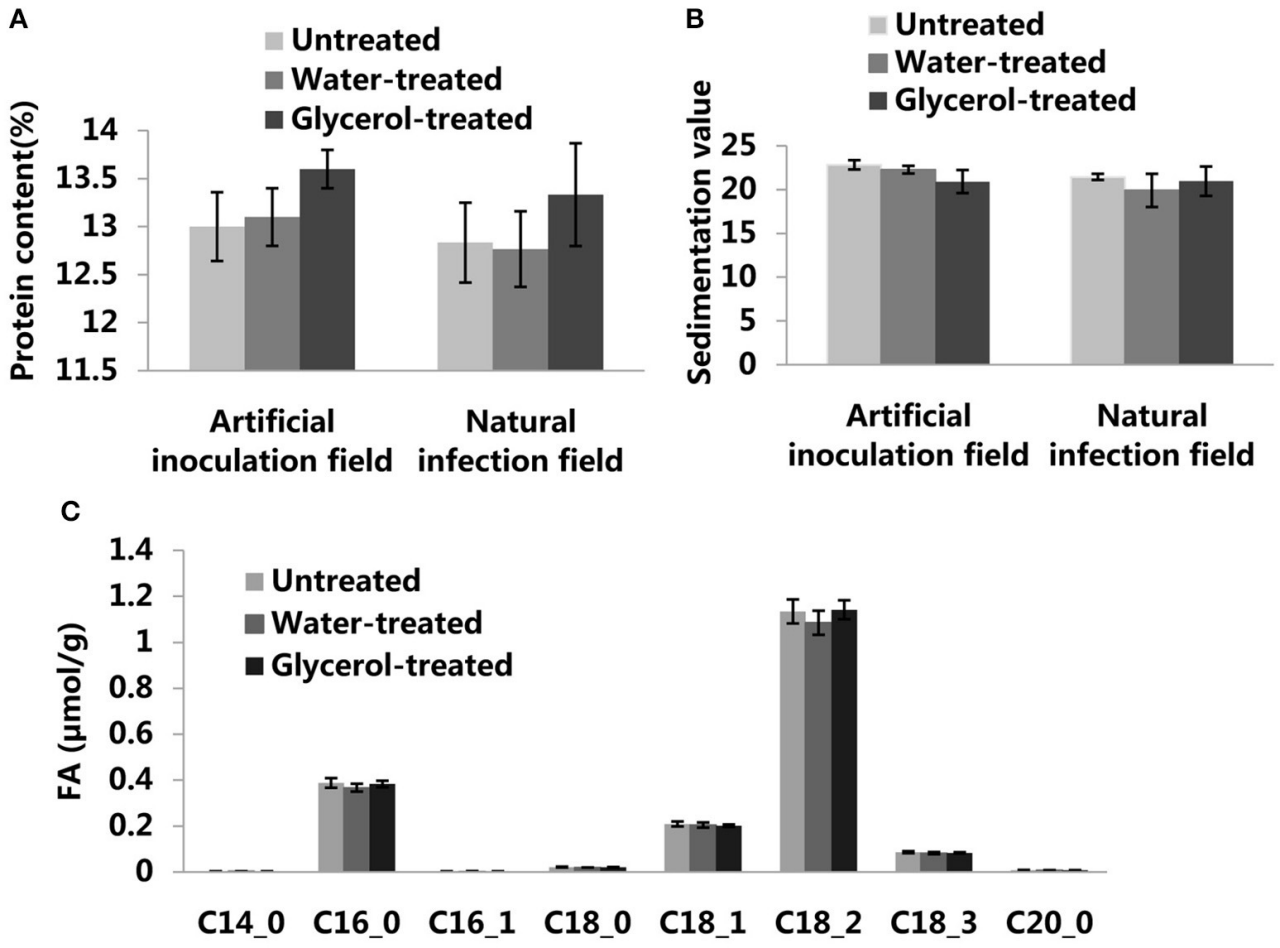
**The effects of glycerol treatment on seed quality. (A)** Protein content of untreated, water-treated, and glycerol-treated Xuezao seeds in the artificial inoculation field and natural infection field. **(B)** SDS value of untreated, water-treated, and glycerol-treated Xuezao plants seeds in the artificial inoculation field and natural infection field. **(C)** FA content of untreated, water-treated, and glycerol-treated Xuezao seeds in the artificial inoculation field. Each value is the mean ± SE of three independent biological repetitions.

In order to assess the potential for the use of glycerol as a foliar spray to control wheat powdery mildew, we continued our research on glycerol application experiments in field conditions in both 2015 and 2016. In artificial inoculation field experiments, *Bgt* isolate E09 and leaf rust isolate PHT were inoculated on plants during the seeding stage. Glycerol or water was sprayed on the plants 4 times (once every 3 days) prior to the outbreak period of powdery mildew disease. We found that the leaves and ears of Xuezao plants were highly susceptible to powdery mildew, both in the untreated and water-treated assays; the glycerol-treated leaves and ears of Xuezao plants were, however, highly resistant to powdery mildew (Figure 6A). In contrast, glycerol treatment had little inhibitory effect on the progression of leaf rust disease (Figure 6A). We found that the glycerol treatment had no effect on grain numbers per ear (Figure S7). Then we measured the TKW (thousand kernel weight) of untreated, water-treated, and glycerol-treated plants. In the artificial inoculation fields, there was little difference in TKW between untreated and water-treated Xuezao plants, but the TKW of glycerol-treated plants was significantly higher, representing a kernel weight increase of more than 15% in the 2015 year and more than 9% in the 2016 year (Figure 6B). We also simulated normal production conditions in a field that was far away from the disease inoculation fields, where the extent of the disease outbreak was not as serious as in the artificial inoculation fields, and only two times glycerol applications were needed to effectively control the disease. The TKW of glycerol-treated plants was significantly higher, representing a kernel weight increase of more than 5% both in the 2015 and 2016 years (Figure 6B). The TKW of the partially-resistant wheat variety Jing411, when treated with glycerol in the artificial inoculation field, was induced resistance and was 5% greater than the TKW for both the untreated and the water-treated plants (Figures S8A,B). We sprayed glycerol on resistant lines 6Y8, 6Y9, 6Y16, and 6Y19 carrying resistant gene *Pm21* in the natural infection field and found that the glycerol treatment did not increase the grain weight (Figure S9). So, we speculated that the increase in grain weight in the susceptible wheat lines wasn't due solely to glycerol treatment. The leaves and ears of Xuezao line were highly susceptible to powdery mildew in the artificial inoculation field. We conducted experiments where we sprayed glycerol on leaves or ears, separately. Result showed that spraying glycerol only on Xuezao leaves did not alter powdery mildew infection of ears, and spraying glycerol only on wheat ears showed high resistance in the Xuezao ears (Figure S10A). The TKW of plants that had only their ears treated with glycerol increased by ~9% compared to untreated plants, and it increased by about 2% in plants that had only their leaves treated with glycerol compared to untreated plants (Figure S10B). The quantity and quality of the gluten in wheat and flour can be estimated by assessing the grain protein content and the SDS sedimentation volume. So, we measured the grain protein content and SDS sedimentation volume of wheat seeds to examine whether the glycerol application affect wheat quality. The grain protein content and SDS sedimentation volume were not significantly changed among the untreated, water-treated, and glycerol-treated plants in the artificial inoculation or in the natural infection field experiments (Figures 7A,B). There were no changes in the FAs levels of wheat seeds among the untreated, water-treated and glycerol-treated plants (Figure 7C). So, glycerol application in the wheat fields significantly reduced the severity of powdery mildew disease and lessened disease-caused kernel weight losses, without causing any noticeable degradation of wheat seed quality.

## Discussion

A previous study in Arabidopsis showed that *gli1* (a glycerol kinase) mutant accumulates a high levels of glycerol and is more susceptible to *C. higginsianum* than wild-type plants (Chanda et al., 2008). Our results showed that glycerol did not induce wheat resistance when applied on the same day as the inoculation of fungus or later (Figure 1B). So, we speculated that glycerol had no direct/toxic effect on powdery mildew, but rather induced plant resistance responses against powdery mildew. We observed that the G3P accumulation level increased after infection with powdery mildew. G3P is important signal molecule in plant immune responses against fungi (Chanda et al., 2011; Yang et al., 2013). So, we surmised that the accumulation of G3P is an active defense process in wheat defense responses against powdery mildew. It has been demonstrated that unsaturated FAs (18:1, 18:2, and 18:3) can inhibit innate immunity-related callose formation (Blümke et al., 2014). Therefore, the higher levels of *TaSSI2* gene expression and oleic acid (18:1), as induced by powdery mildew-infection, were likely to benefit fungi infection. This might not be involved in the plant defense responses against powdery mildew, and was perhaps just the passive response in wheat following powdery mildew infection. Instead, low level of oleic acid can induce disease resistance in plants (Kachroo et al., 2001, 2004; Song et al., 2013). Glycerol application not only increased G3P levels, but also reduced the oleic acid level, thereby contributing to resistance in wheat.

Previous studies have demonstrated that *GLY1*-encoded G3Pdh plays an important role in Arabidopsis basal resistance to *C. higginsianum*, as *gly1* plants were much more susceptible than the *gli1* plants (Chanda et al., 2008). The expression of *TaGLY1* was likewise significantly induced by avirulent *Pst* infection (Yang et al., 2013). Our study indicated that *TaGLY1* expression was not induced by powdery mildew-infection. The expression of *TaGLI1* and *TaSSI2* in the glycerol-treated leaves was reduced compared to that of the water-treated leaves after infection with powdery mildew (Figures 2A, 3). There are two potential explanations for this. One is that glycerol application induced wheat resistance against powdery mildew and slowed the infection, and gene expression responses to powdery mildew were weakened. The other explanation may be that glycerol application increased G3P levels and slowed the expression of *TaGLI1* through a feedback repression system. We also checked the expression patterns of the predicted barley homologous genes of *SSI2, GLI1*, and *GLY1* in PLEXdb (Plant Expression Database), and found that the expression of *HvSSI2* (GenBank: AK353961.1) and *HvGLI1* (GenBank: AK252700.1) was induced by various powdery mildew isolates in different barley genotypes, while the expression of the *HvGLY1* (Probe Set ID: Contig 23957) gene was down-regulated post-infection (Figures S11–S13; Caldo et al., 2004). We speculate that the role of the *SSI2* and *GLI1* genes in the powdery mildew-infection might be conserved in some triticeae crops.

Strong ROS accumulation (e.g., H_2_O_2_) and SA play an important role in the effective formation of papillae as a general wheat defense against powdery mildew. HR responses are associated with high H_2_O_2_ accumulation (Li et al., 2005). Glycerol application induced the expression of *pathogenesis-related* (*PR*) genes, and induced the generation of ROS and SA before powdery mildew infection (Figures 5A–C), and this might have initiated some immune responses ahead of pathogen-infection and contributed to the resistance observed in the susceptible wheat lines. G3P, as a critical immune signal, is generated following local *C. higginsianum* infection and translocated to distal tissues and confer SAR in Arabidopsis (Chanda et al., 2011). However, in our study, we found that glycerol application induced only local resistance in wheat, and it was not induced resistance in the whole plant with localized glycerol application (Figure 5D, Figures S6, S10A), This is consistent with the reported result that exogenous application of glycerol in cacao local leaves does not induce resistance responses in distal leaves (Zhang et al., 2015). It remains further study on the mechanism of the glycerol-induced local defense response in wheat.

Wheat powdery mildew is a devastating worldwide disease that is capable of greatly reducing wheat yield (Griffey et al., 1993). A low-cost and effective strategy for disease control is thus greatly needed. Glycerol is an environmentally-friendly, non-toxic, edible, and biodegradable sugar alcohol chemical that is generated in excess as a low-cost byproduct from the biodiesel industry (Yang et al., 2012). In addition, wheat seeds germinated in 2.5–3% glycerol showed resistance in the seedling stage, suggesting that glycerol could be used for irrigating or as a seed-treatment material. It is demonstrated that glycerol treatment alters endogenous levels of G3P, phosphate and ROS, affects auxin distribution and cell division in the root meristem, and eventually results in inhibition of primary root growth in Arabidopsis (Hu et al., 2014). Further studies will be needed to explore the potential of deleterious inhibition of wheat growth caused by glycerol application at the seeding stage. However, we sprayed glycerol in a wheat field and found that it significantly reduced the severity of powdery mildew disease and lessened disease-associated kernel weight loss, all without causing any noticeable degradation in wheat seed quality. It is worth noting that glycerol is suitable for use as a cosolvent material in the application of agrochemicals. Above all, glycerol appears to have some potential to be applied in the field as an environmentally-friendly agricultural chemical to help manage plant diseases, however, the frequency of spraying may limit its use in large scale wheat production.

## Author contributions

CX, QS, and YL conceived the project. YL and MG collected the plant materials, YL, FL, and NS performed the experiments. YL, WL, YW, and CZ analyzed data. YL wrote the manuscript.

### Conflict of interest statement

The authors declare that the research was conducted in the absence of any commercial or financial relationships that could be construed as a potential conflict of interest.
