# Supplementary material for: Application of Glycerol for Induced Powdery Mildew Resistance in Triticum aestivum L
Source: Front Physiol. 2016 Sep 21;7:413. doi: 10.3389/fphys.2016.00413 (PMC5030236; doi:10.3389/fphys.2016.00413)
Supplement: Supplementary file 1 [file Table1.DOCX]

**Table S1.** Primer pairs used in this study**.**

| **Primer Names** | **Sequence** | **Target genes** |
| --- | --- | --- |
| *Actin-QF* | TACTCCCTCACAACAACCG | qPCR primers for *Actin* |
| *Actin-QR* | AGAACCTCCACTGAGAACAA |  |
| *TaGLI1-QF* | TCAAGCAGCACTACCCG | qPCR primers for *TaGLI1* |
| *TaGLI1-QR* | CAAACCAGCATCCACATTA |  |
| *TaGLY1-QF* | GGAGCAAAGCCAACAACCC | qPCR primers for *TaGLY1* |
| *TaGLY1-QR* | ACCAAGACGCAATCCCACA |  |
| *TaSSI2-QF* | CGGACTCCCATAAACTG | qPCR primers for *TaSSI2* |
| *TaSSI2-QR* | ACGGTCGACTCACTCAC |  |
| *TaPR1-QF* | TGCACGTTCCTATCTACTG | qPCR primers for *TaPR1* |
| *TaPR1-QR* | CAAGGTACCGTAAAAATGCACA |  |
| *TaPR2-QF* | AACGACCAGCTCTCCAACAT | qPCR primers for *TaPR2* |
| *TaPR2-QR* | GTATGGCCGGACATTGTTCT |  |
| *TaPR3-QF* | ACGGTGTGATCACCAACATC | qPCR primers for *TaPR3* |
| *TaPR3-QR* | CAGTCCAGGTTGTCACCGTA |  |
| *TaPR4-QF* | AAAGAACGAGACGCTCCCA | qPCR primers for *TaPR4* |
| *TaPR4-QR* | CCGCTGTTACAACTTACAACC |  |
| *TaPR5-QF* | GACTTCTACGACATCTCGGTG | qPCR primers for *TaPR5* |
| *TaPR5-QR* | GGTAGTTACTGTTGCCATTGC |  |
| *TaGLI1-F* | CTTGTTCCACCGGCTCACTC | primers for *TaGLI1*gene cloning |
| *TaGLI1-R* | *CTTTCTGAAGAATCCAGATGTTCCT* |  |
