# Supplementary material for: Application of Glycerol for Induced Powdery Mildew Resistance in Triticum aestivum L
Source: Front Physiol. 2016 Sep 21;7:413. doi: 10.3389/fphys.2016.00413 (PMC5030236; doi:10.3389/fphys.2016.00413)
Supplement: Supplementary file 2 [file Table2.DOCX]

**Table S2** The germination and penetration rates of powdery mildew in untreated, water-treated and glycerol-treated leaves at 12, 24, 48, and 96 hours post infection.

| **Time (post powdery mildew infection)** | **Untreated** | | **Water-treated** | | **Glycerol-treated** | |
| --- | --- | --- | --- | --- | --- | --- |
|  | Germination rate (%) | Penetration rate (%) | Germination rate (%) | Penetration rate (%) | Germination rate (%) | Penetration rate (%) |
| 12 hour | 87.8±3.2 | 0 | 88.7±1.6 | 0 | 12.4±2.3** | 0 |
| 24 hour | 95.6±2.1 | 0 | 94.9±11.1 | 0 | 16.3±0.8** | 0 |
| 48 hour | 92.9±3.3 | 67.8±7.7 | 91.7±15.6 | 70.4±6.6 | 19.2±5.3** | 5.9±3.2** |
| 96 hour | 92.4±2.0 | 85.6±6.6 | 89.8±5.0 | 81.2±7.0 | 31.4±2.3** | 8.9±1.9** |

Note: Germination rates (number of germinated and penetrations pores relative to the total number of spores); Penetration rates (number of penetrations pores relative to the total number of spores). Each value is the mean ± SE of three independent biological repetitions. Asterisks (**) indicate significant difference from untreated type at P < 0.01 by Student's t-test. The phenotype of germinated and penetrations pores was showed in **figure 1E**.
