# Supplementary material for: Application of Glycerol for Induced Powdery Mildew Resistance in Triticum aestivum L
Source: Front Physiol. 2016 Sep 21;7:413. doi: 10.3389/fphys.2016.00413 (PMC5030236; doi:10.3389/fphys.2016.00413)
Supplement: Supplementary file 3 [file Image13.PDF]

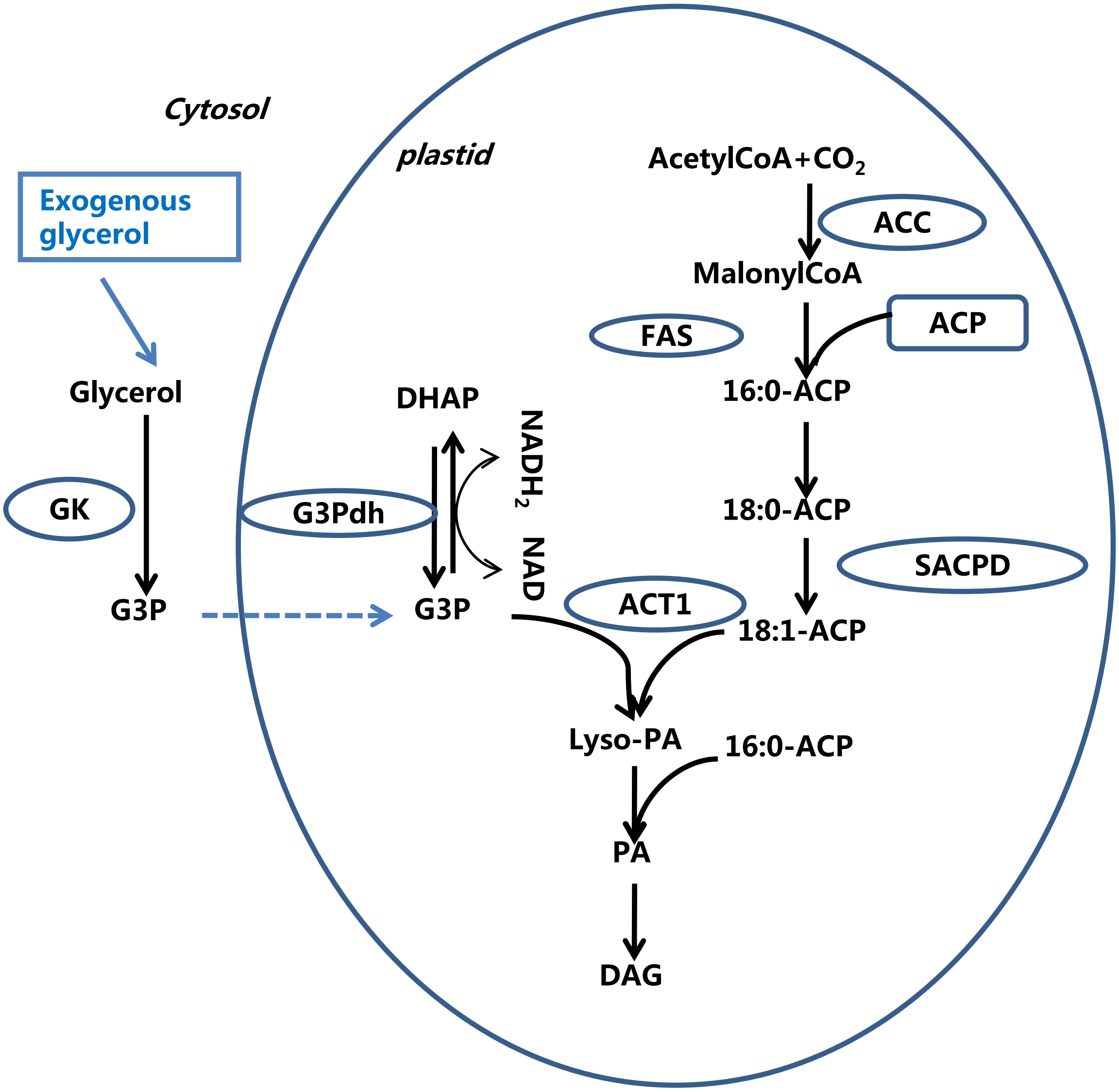

Figure S1. A condensed scheme of plastid fatty acid biosynthesis in plants. Abbreviations used are: ACC, AcetylCoA carboxylase; FAS: Fatty acids; ACP, Acyl carrier protein; PA, phosphatidic acid; DAG, diacylglycerol; GK, glycerolipid; G3Pdh, glycerophosphate dehydrogenase. The acylation of G3P is catalyzed by the ACT1-encoded G3P acyltransferase (adapted from Kachroo et al., 2009).

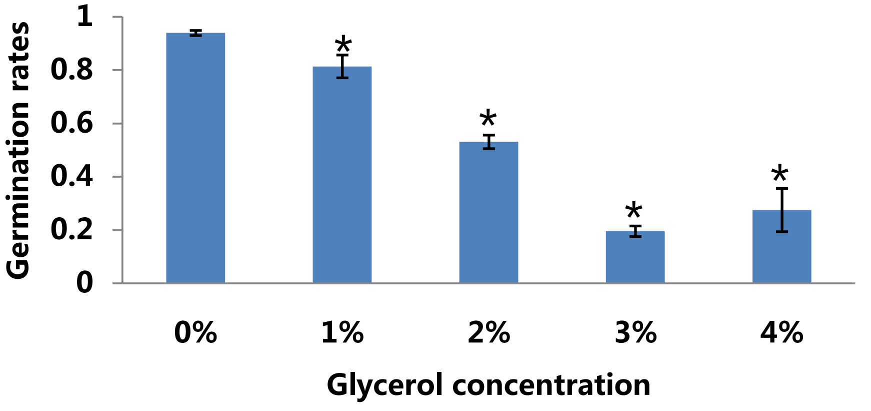

Figure S2. Germination rates of powdery mildew on wheat leaves at 48 hpi with a series of concentrations (0%, 1%, 2%, 3%, and 4%) of glycerol solutions. The glycerol pretreatments (one time) were employed one day before infection with powdery mildew. Each value is the mean ± SE of three independent biological repetitions, and 15-20 leaves were observed in each independent biological experiment. Asterisks indicate significant differences from the germination rates of 0% glycerol application at $P < 0.05$ by Student's t-test.

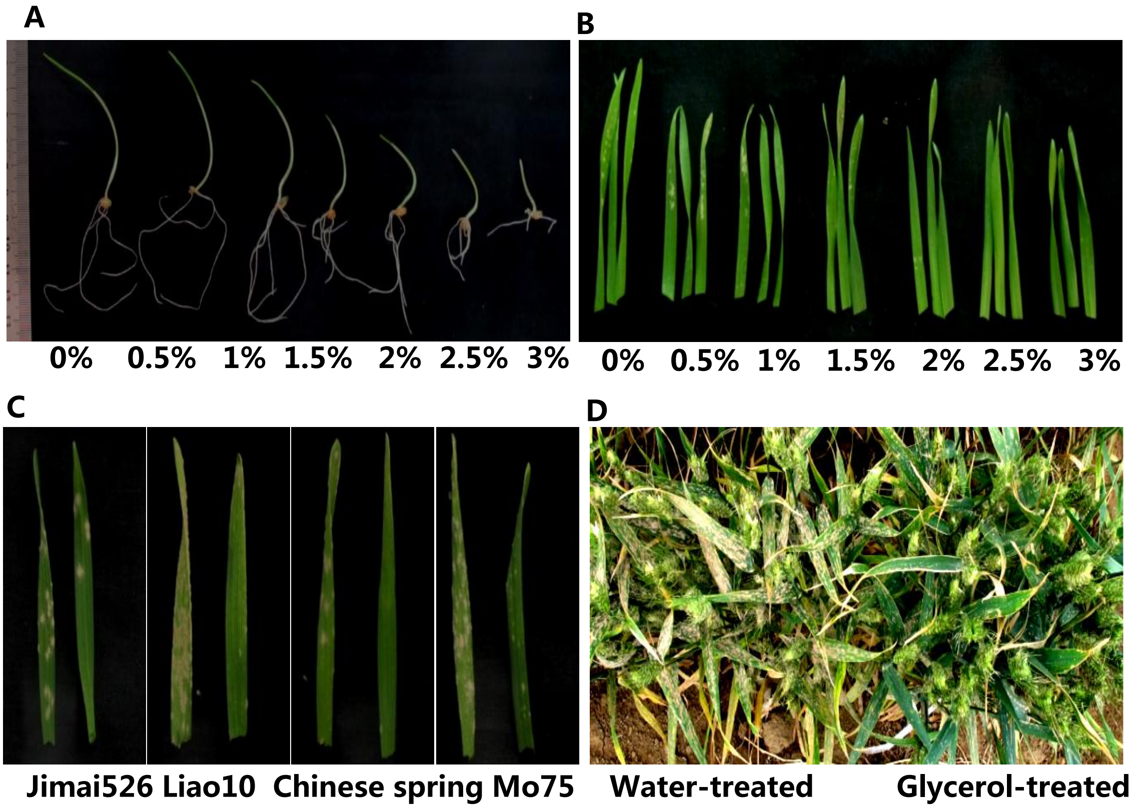

Figure S3. Glycerol application enhanced powdery mildew resistance in wheat. **(A, B)** morphology and resistance phenotypes of Xuezao plants after seed germination following treatment with 0%, 0.5%, 1%, 1.5%, 2%, 2.5%, and 3% glycerol solutions. The 0-3% glycerol prepared in sterile water for germination of wheat seeds, after five days germination planted them into nutrient soil and inoculated powdery mildew. The resistance phenotypes of plants were observed after one weeks post powdery mildew infection. (C) Resistance phenotypes in several wheat varieties after glycerol application. Left leaf: Water-treated leaf; Right leaf: Glycerol-treated leaf. (D) Images of water-treated and glycerol-treated Xuezao adult plants at two weeks post powdery mildew-infection. Each of the experiments were conducted with three independent biological repetition; each independent biological repetitions included twenty plants.

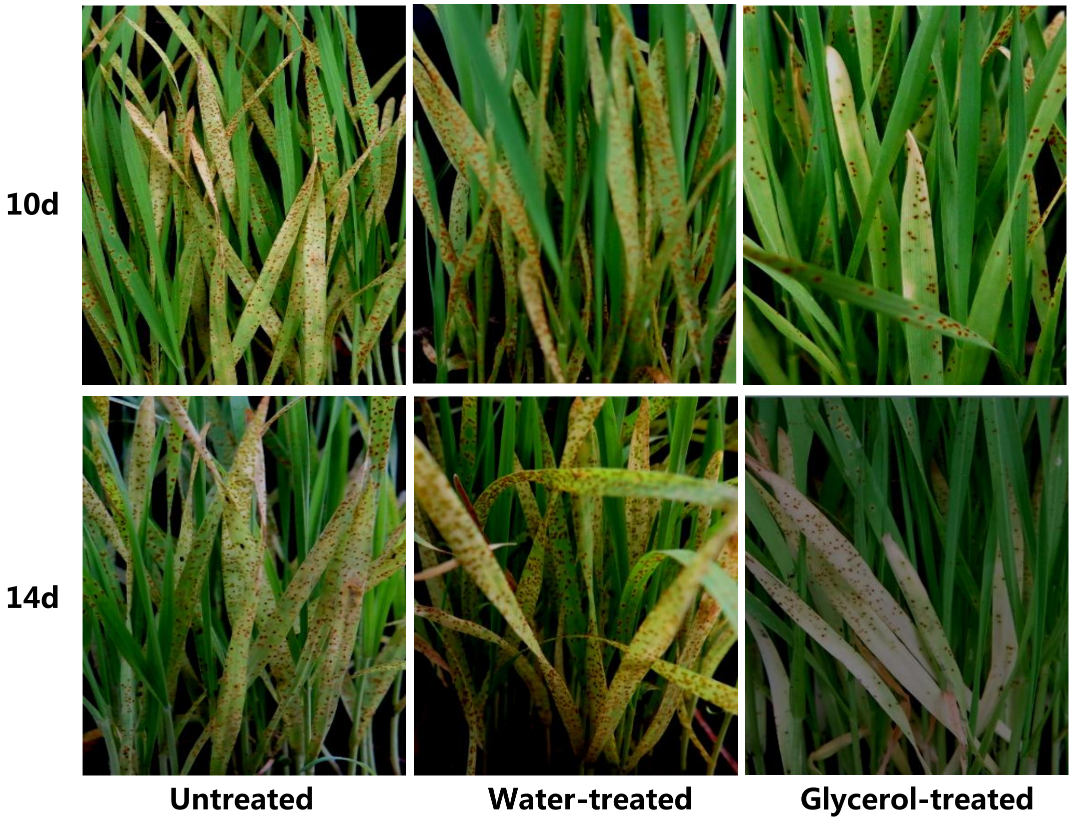

Figure S4. Images of untreated, water-treated, and glycerol-treated Xuezao leaves at 10 days and 14 days post leaf rust infection**.**

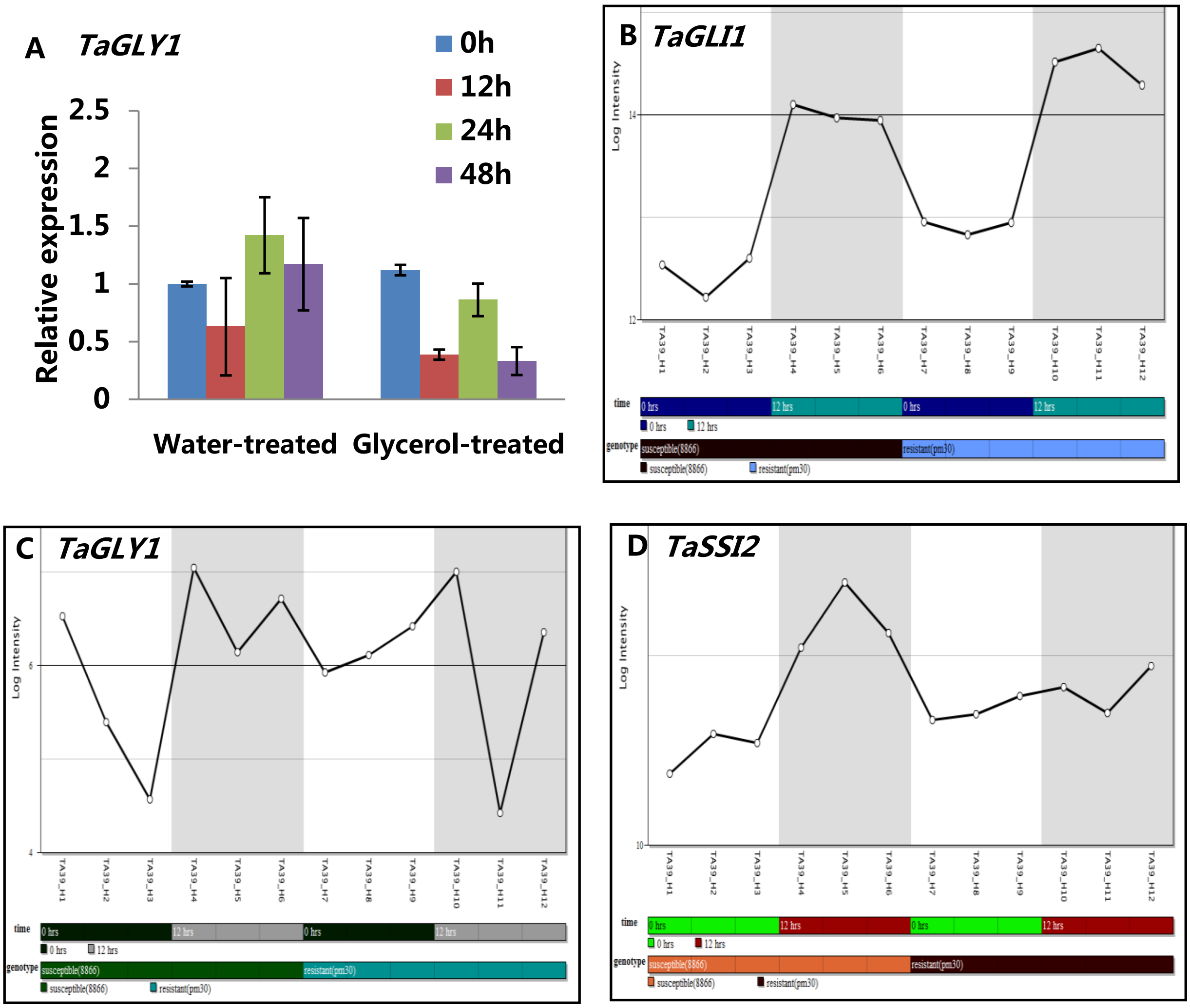

Figure S5. Relative transcriptional changes of the *TaGLY*, *TaGLI1*, and *TaSSI2* genes in response to powdery mildew. (A) Relative transcriptional changes of *TaGLY* both in the water-treated (control) and glycerol-treated leaves at 0, 12, 24, and 48 hours post powdery mildew-infection (hpi). Each value is the mean ± SE of three independent biological repetitions. (B, C, and D) Expression patterns of *TaGLI1*, *TaGLY1,* and *TaSSI2* in susceptible line JD8 and its powdery mildew-resistant near-isogenic resistant line Pm30 from our previous microarray study (Xin et al., 2011).

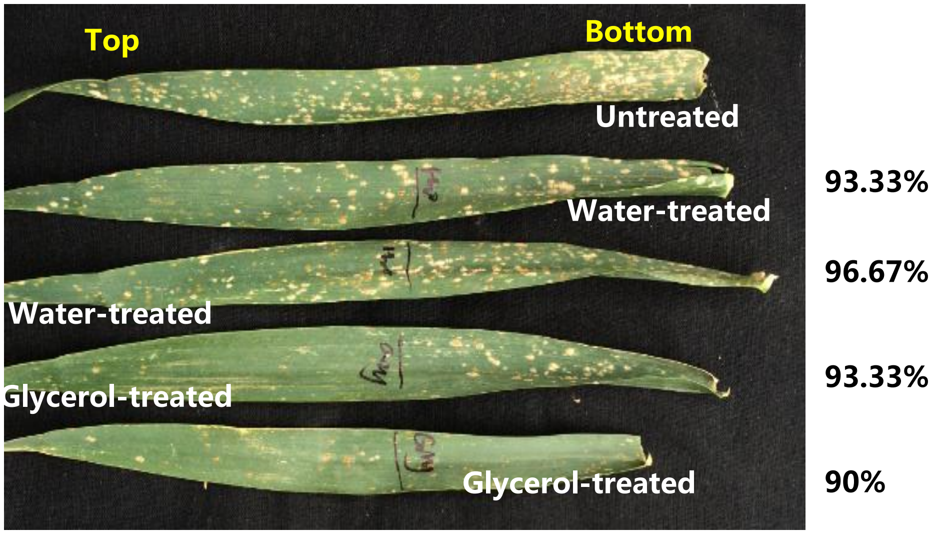

Figure S6. Glycerol application induced local resistance in wheat. Images of the top or the bottom half of Xuezao leaf leaves with water or glycerol treatments in the artificial inoculation field. 30 leaves were observed the experiment, and the percentages of resistance phenotypes showed in the images were 93.33%, 96.67%, 93.33%, and 90%.

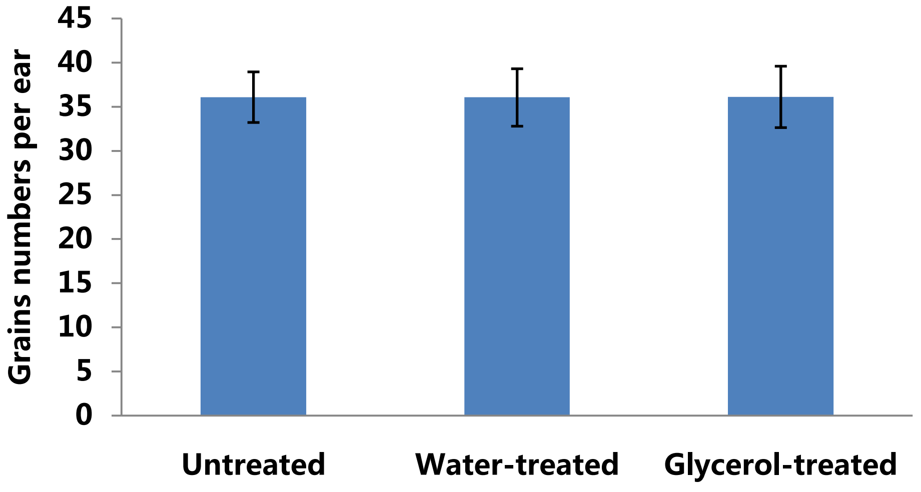

Figure S7. Grain numbers per ear of untreated, water-treated, and glycerol-treated Xuezao plants in the artificial inoculation field. Each value is the mean ± SE of 30 ears observed in the experiment.

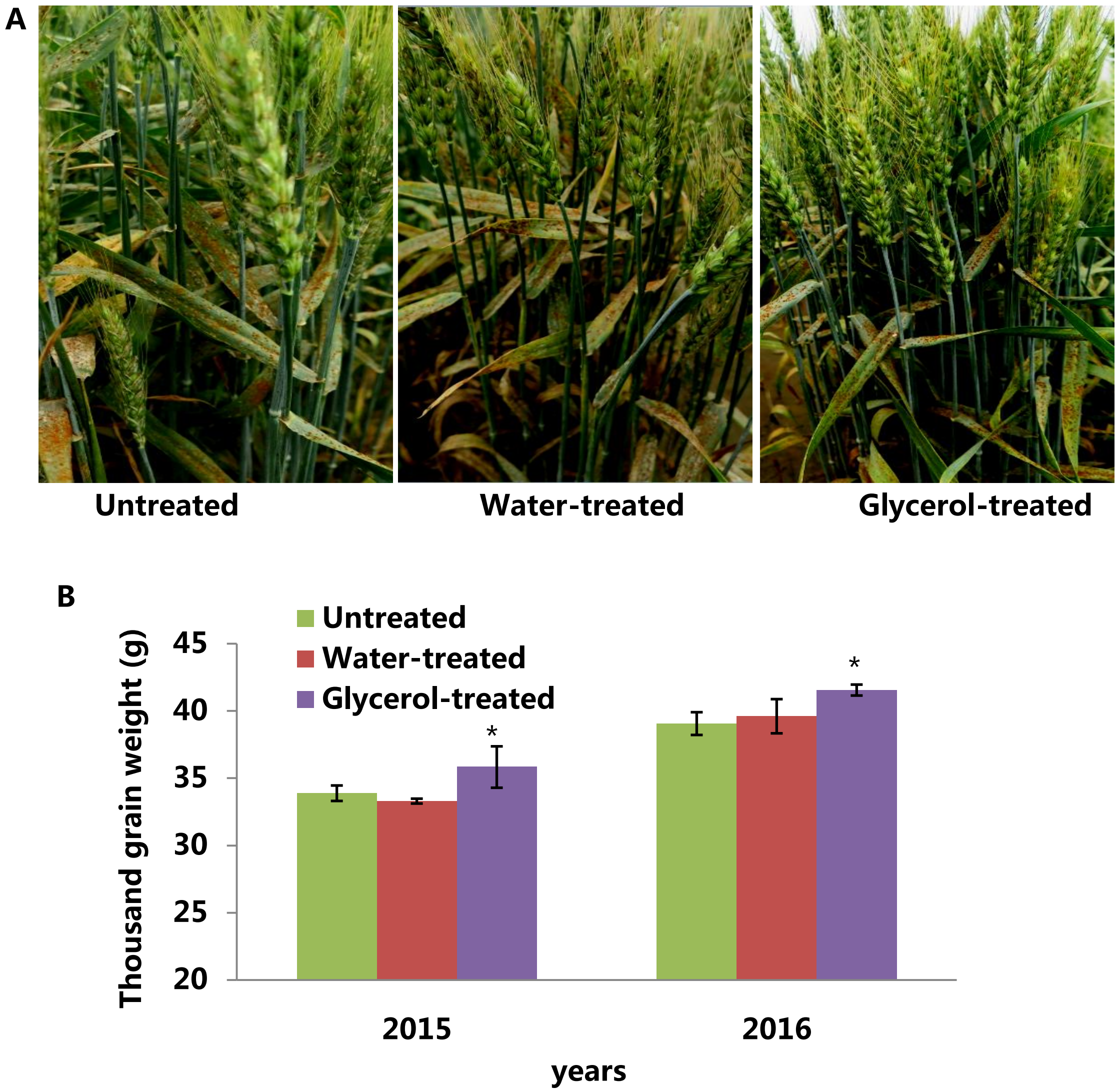

Figure S8. The potential of glycerol application as a foliar spray in field. (A) Images of untreated, water-treated, and glycerol-treated Jing 411 (partially resistant to wheat powdery mildew) leaves and ears in the artificial inoculation field. (B) The thousand kernel weight of untreated, water-treated, and glycerol-treated Jing 411 in the artificial inoculation field. Each value is the mean ± SE of three independent biological repetitions. Asterisks indicate significant differences from untreated samples at $P < 0.05$ by Student's t-test.

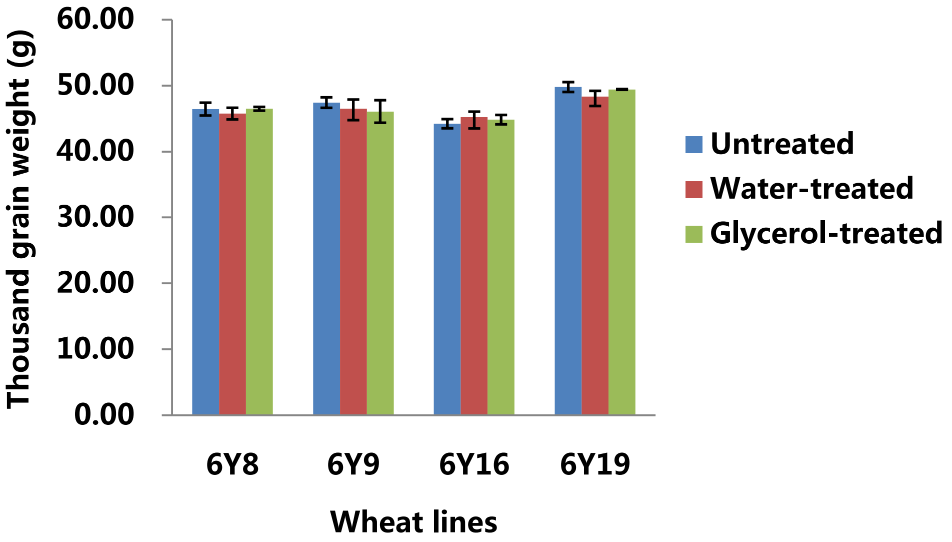

Figure S9. (A) The thousand kernel weight of untreated, water-treated, and glycerol-treated wheat resistant lines 6Y8, 6Y9, 6Y16, and 6Y19 carrying the resistance gene *Pm21* in the natural infection field. Each value is the mean ± SE of three independent biological repetitions.

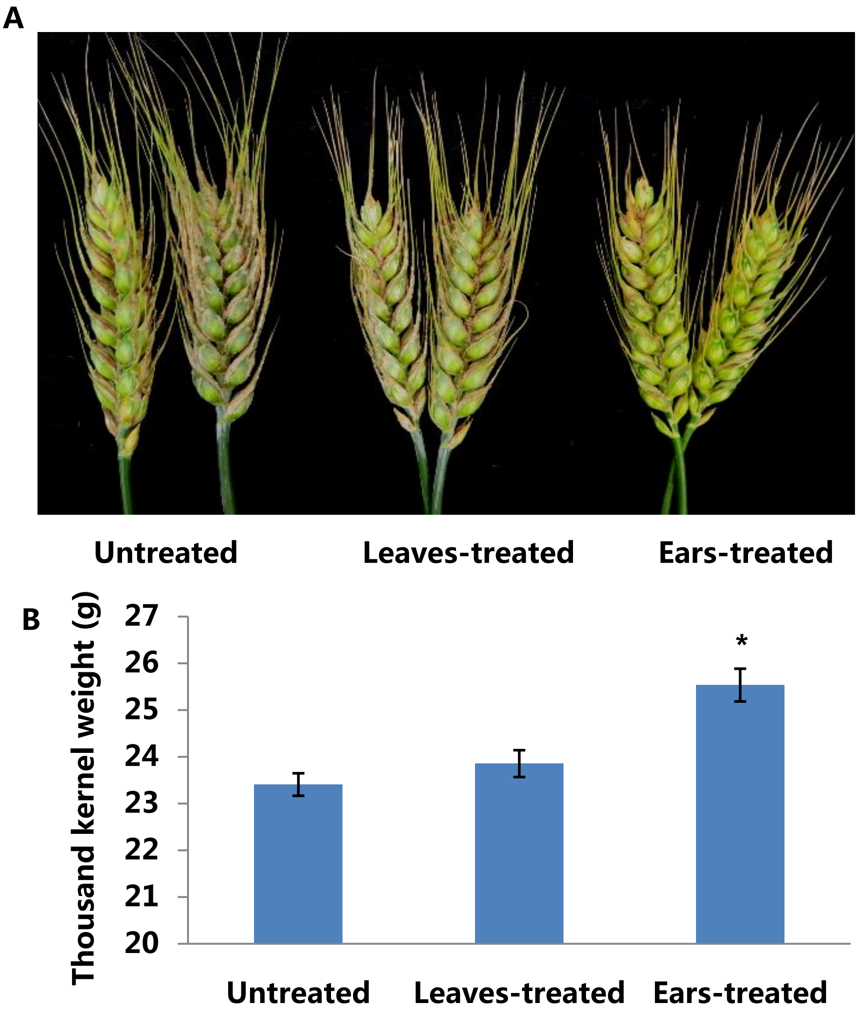

Figure S10. The effect of glycerol application on leaves or ears, separately, on resistance and thousand kernel, in Xuezao plants. (A) Images of Xuezao ears from untreated, leaf-treated, and ear-treated (with glycerol) plants the artificial inoculation field. (B) The thousand kernel weight of Xuezao plants of untreated, leaf-treated, and ear-treated (with glycerol) in the artificial inoculation field. (Untreated) Without any treatments; (Leaves-treated) Spraying glycerol only on wheat leaves; (Ears-treated) Spraying glycerol only on wheat ears. Each value is the mean ± SE of three independent biological repetitions. Asterisks indicate significant differences from the untreated samples at $P < 0.05$ by Student's t-test.

HvGLI1

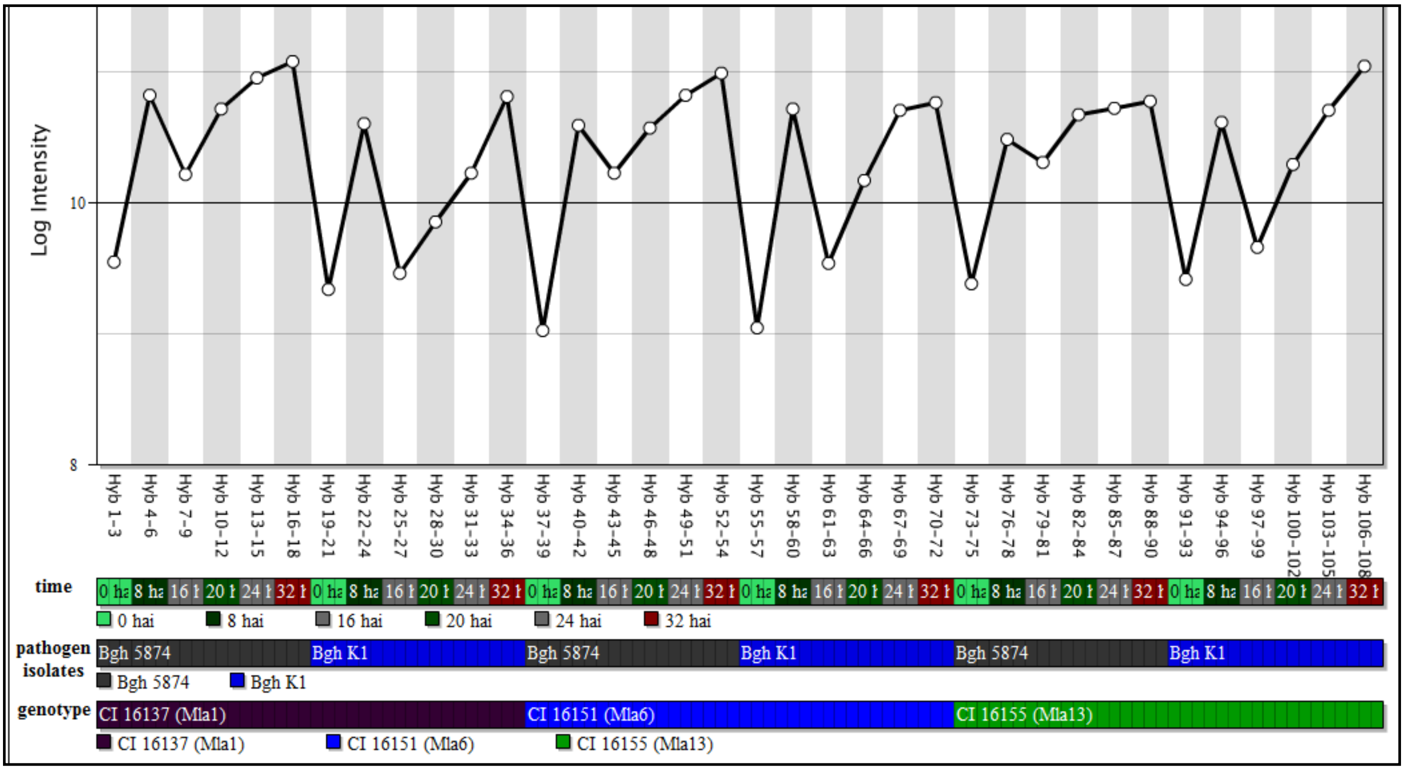

Figure S11. The expression pattern of the *HvGLI1* gene in different barley genetypes in response to interaction with various powdery mildew isolates from a previous microarray study (Caldo et al., 2004).

HvSSI2

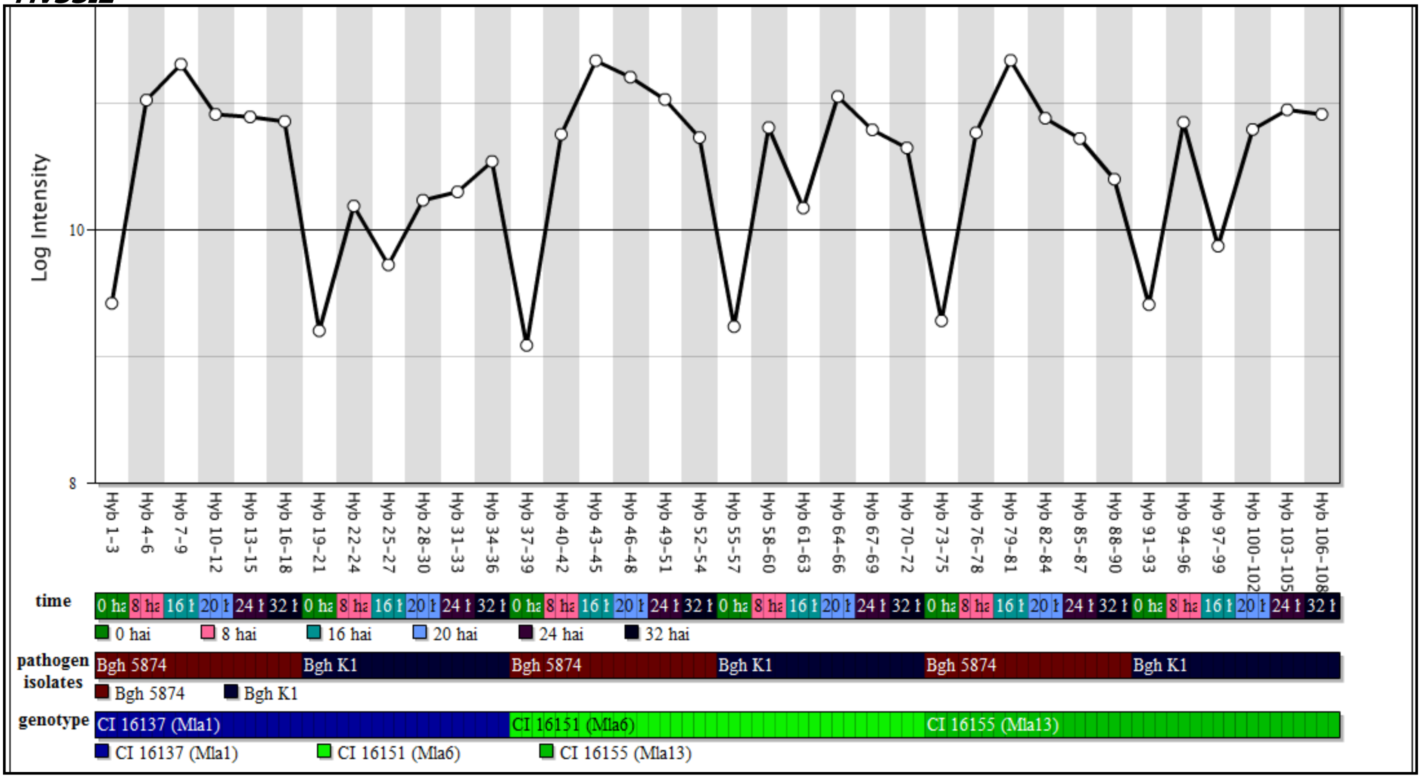

Figure S12. Expression patterns of the *HvSSI2* gene in different barley genetypes in response to interaction with various powdery mildew isolates from a previous microarray study (Caldo et al., 2004).

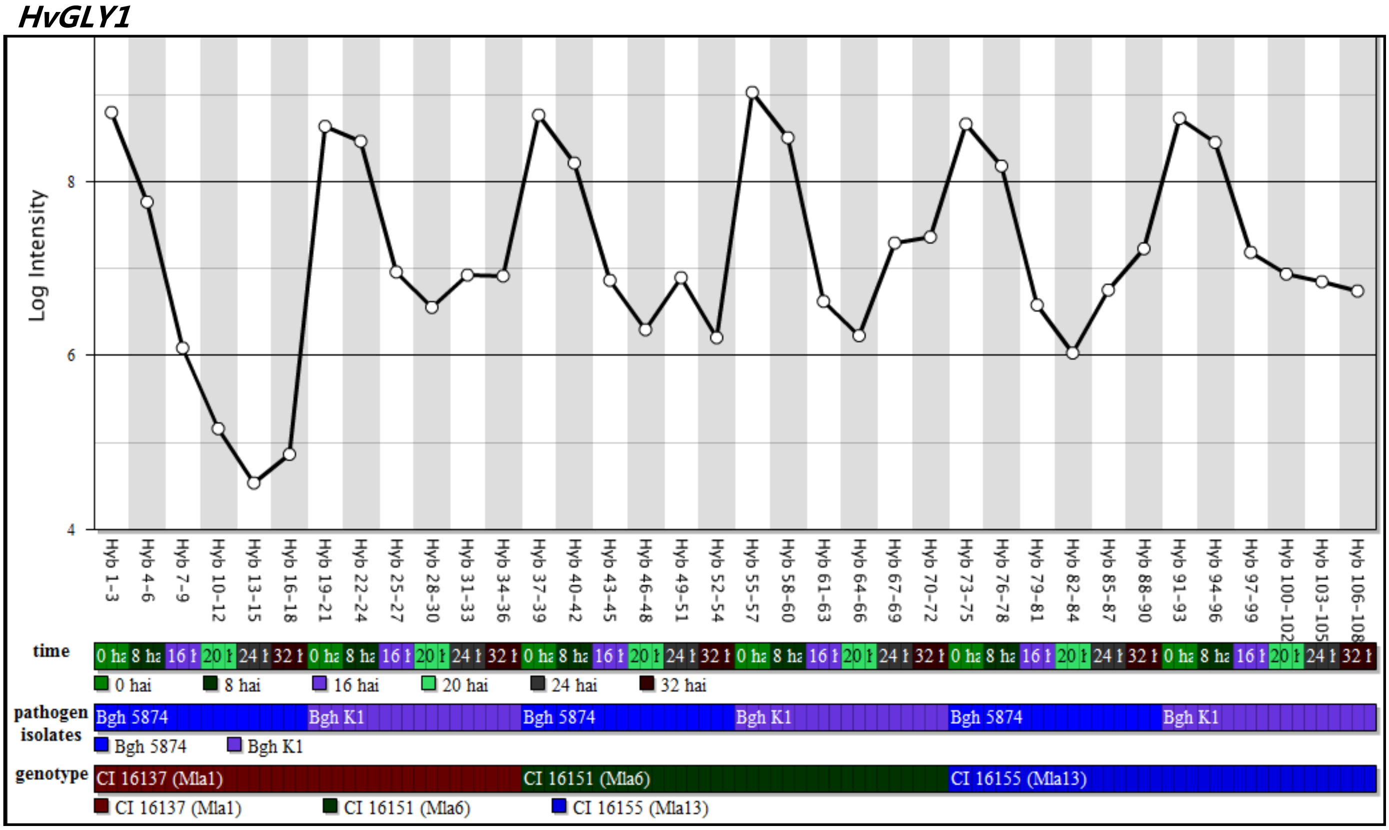

Figure S13. Expression patterns of the *HvGLY1* gene in different barley genetypes in response to interaction with various powdery mildew isolates from a previous microarray study (Caldo et al., 2004).
